# DNA methylation enables recurrent endogenization of giant viruses in an animal relative

**DOI:** 10.1126/sciadv.ado6406

**Published:** 2024-07-12

**Authors:** Luke A. Sarre, Iana V. Kim, Vladimir Ovchinnikov, Marine Olivetta, Hiroshi Suga, Omaya Dudin, Arnau Sebé-Pedrós, Alex de Mendoza

**Affiliations:** ^1^ School of Biological and Behavioural Sciences, Queen Mary University of London, London, UK.; ^2^ CRG, Centre for Genomic Regulation (CRG), Barcelona Institute of Science and Technology (BIST), Barcelona, Spain.; ^3^ Swiss Institute for Experimental Cancer Research, School of Life Sciences, Swiss Federal Institute of Technology (EPFL), Lausanne, Switzerland.; ^4^ Faculty of Life and Environmental Sciences, Prefectural University of Hiroshima, Shobara, Japan.; ^5^ ICREA, Barcelona, Spain.; ^6^ Universitat Pompeu Fabra (UPF), Barcelona, Spain.

## Abstract

5-Methylcytosine (5mC) is a widespread silencing mechanism that controls genomic parasites. In eukaryotes, 5mC has gained complex roles in gene regulation beyond parasite control, yet 5mC has also been lost in many lineages. The causes for 5mC retention and its genomic consequences are still poorly understood. Here, we show that the protist closely related to animals *Amoebidium appalachense* features both transposon and gene body methylation, a pattern reminiscent of invertebrates and plants. Unexpectedly, hypermethylated genomic regions in *Amoebidium* derive from viral insertions, including hundreds of endogenized giant viruses, contributing 14% of the proteome. Using a combination of inhibitors and genomic assays, we demonstrate that 5mC silences these giant virus insertions. Moreover, alternative *Amoebidium* isolates show polymorphic giant virus insertions, highlighting a dynamic process of infection, endogenization, and purging. Our results indicate that 5mC is critical for the controlled coexistence of newly acquired viral DNA into eukaryotic genomes, making *Amoebidium* a unique model to understand the hybrid origins of eukaryotic DNA.

## Teaser

The protist *Amoebidium* genome illuminates animal DNA methylation evolution and tolerance to giant virus integration in eukaryotes.

## INTRODUCTION

5-Methylcytosine (5mC) is a common base modification among eukaryotes (*1*–*3*). 5mC is deposited by DNA methyltransferases (DNMTs), a family of enzymes with ancestral families conserved throughout eukaryotes (*4*, *5*). Some DNMTs are maintenance type enzymes, perpetuating 5mC patterns, including DNMT1 and DNMT5, while other DNMTs have de novo activity, such as DNMT3 (*6*, *7*). However, the DNMT repertoire of an organism is not predictive of 5mC function. In some eukaryotes, including plants and animals, 5mC is associated with gene regulation, exemplified by gene body methylation, where 5mC positively correlates with gene transcriptional levels (*1*, *3*, *8*). However, the most widespread role of 5mC is in transposable element (TE) silencing, which is the assumed ancestral role in eukaryotes (*9*, *10*).

Despite most attention being devoted to controlling endogenous parasitic elements, one of the first described functions of 5mC in eukaryotes was to silence retroviral insertions in mammals (*11*). Similarly, in bacteria, the main role of 5mC is to combat viruses (*12*). Therefore, controlling exogenous viral invasions is arguably as important as TE control for epigenetic silencing. It is increasingly recognized that many eukaryotic genes have viral origins, co-opted repeatedly throughout evolution (*13*). One of the most common sources for these acquisitions are giant viruses (*Nucleocytoviricota*). Giant viruses have a wide range of eukaryotic hosts and are present in almost all ecosystems, posing a widespread threat to eukaryotic cells (*14*, *15*). Giant viruses are exceptional among viruses as they have enormous genomes (100 kb to 2.5 Mb) encoding many proteins thought to be eukaryotic hallmarks such as histones (*14*, *15*). Giant viruses originated before modern eukaryotes, and they have been proposed to have contributed essential genes to eukaryogenesis (*16*–*18*). Furthermore, recent reports indicate that giant viruses can endogenize into extant eukaryotes (*19*–*21*). However, how this potentially lethal DNA is incorporated into eukaryotic genomes is currently not understood.

**Fig. 1.**
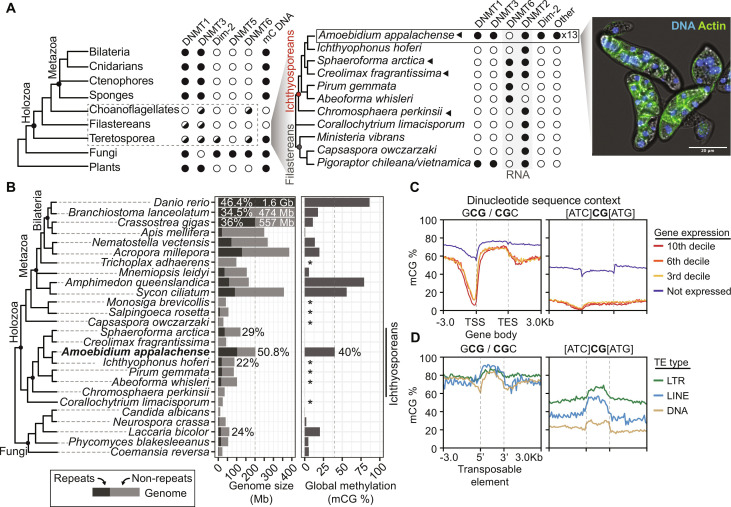
*A. appalachense* displays a methylome with gene body and transposon methylation. (**A**) Distribution of DNMTs in eukaryotes and teretosporeans. Black dot indicates enzyme presence, white dot indicates enzyme absence, and black triangles indicate species for which Enzymatic Methyl-seq has been performed. *Amoebidium* cells are stained with phalloidin and Hoechst. Phylogenetic relationships based on previous studies (*29*). (**B**) Distribution of genome sizes, repetitive content, and global methylation levels across opisthokonts. Asterisks indicate species for which the lack of 5mC is inferred from their absence of DNMTs. Genome sizes for species with genomes above 400 Mb are displayed in white, and repeat content (%) is also highlighted for clarity. (**C**) Gene body methylation and (**D**) TE methylation averages split by the extended CG sequence context. Not expressed genes display <1 TPM. TSS, transcriptional start site; TES, transcriptional end site. TEs only include copies spanning at least 70% of the RepeatModeler2 consensus model.

Finding a link between viral control and epigenetic regulation, however, is hampered by the scarcity of reported recent giant virus endogenizations (*19*, *22*). Moreover, 5mC is evolutionarily very plastic, and many eukaryotic lineages have lost this epigenetic modification (*1*, *2*), possibly because of its mutagenic potential and cytotoxic off-target effects of DNMTs (*23*). Furthermore, 5mC function varies across lineages. In fungi, 5mC is restricted to silencing TEs (*24*), whereas in invertebrates 5mC is usually restricted to gene bodies, and most TEs remain unmethylated (*1*, *2*, *25*). To expand our knowledge of 5mC systems and to unravel how a potentially ancestral fungal-like methylation pattern gave rise to the animal 5mC system, we focused on protists of the holozoan clade. These close animal relatives form four major lineages: choanoflagellates, filastereans, ichthyosporeans, and pluriformeans (Fig. 1A) (*26*, *27*). In recent years, unicellular holozoan genomes have been shown to encode many genes previously thought to be unique to animals, informing the complex genomic nature of the unicellular ancestors of animals (*26*–*29*). However, none of these genomes encode DNMTs, suggesting an evolutionary loss of 5mC capacity (*30*). Here, we fill this gap by describing a unicellular relative of animals that has maintained 5mC, and unexpectedly find an unappreciated and potentially ancestral use of 5mC in regulating giant virus endogenizations.

## RESULTS

### The *Amoebidium* genome presents both gene body and TE methylation

To reconstruct the pre-animal roots of 5mC, we searched the available genomes and transcriptomes of unicellular holozoans for DNMT1 orthologs (*29*, *31*), the maintenance DNMT in animals. We discovered that DNMT1 is expressed by *Amoebidium appalachense* (Fig. 1A), an ichthysoporean originally isolated from the cuticle of freshwater arthropods that can be grown axenically (*32*). Like other Ichthyosporea (*33*), *Amoebidium* are parasites or commensals of animal hosts, and show a coenocytic life cycle, with cells dividing their nuclei in a shared cytoplasm until forming a mature colony, which gives rise to unicellular and uninucleated cells (*34*). Yet, *Amoebidium* branches quite deep within the ichthyosporean lineage (Fig. 1A). *Amoebidium* DNMT1 sequence presents the same domain architecture of animal DNMT1 orthologs, including a zinc finger CXXC absent in non-holozoan sequences (fig. S1A), which suggests that this domain architecture was an innovation of holozoans despite DNMT1 being lost repeatedly. Additionally, we found DNMT1 orthologs in the metagenome-derived assemblies of the filastereans of the genus *Pigoraptor* (*35*), with the same domain architectures as *Amoebidium* (fig. S1A). Additionally, both *Amoebidium* and *Pigoraptor* encode UHRF1 orthologs with highly similar domain architectures to animal counterparts (fig. S1A), suggesting that DNMT1 and UHRF1 heterodimerization is conserved. Unlike *Amoebidium*, *Pigoraptor* species can only be grown at low densities in association with diverse bacteria and eukaryotic prey, the kinetoplastid *Parabodo caudatus*, making functional approaches and reliable methylation profiling challenging (*36*).

To fully characterize the gene repertoire and investigate *Amoebidium* 5mC patterns, we sequenced the genome of this species using a combination of nanopore long reads, Illumina short reads, and micro-C. We obtained a chromosome-scale assembly spanning 202 Mb, with 96.6% of the sequence contained in 18 chromosomes and a 95% BUSCO score (Fig. 1B and fig. S2, A and B). When examining *Amoebidium*’s DNMT repertoire, we identified a total of 18 DNMTs, comprising orthologs of DNMT1, DNMT3, DNMT2, Dim-2, and various lineage-specific clades (Fig. 1A and fig. S1, B and C). Notably, unlike DNMT1, none of the other DNMTs exhibit additional domains. Specifically, DNMT3 lacks the protein domains found in animal DNMT3, such as PWWP or ADD (fig. S1A) (*7*, *30*). In contrast, none of the ichthyosporeans with genomic data available show DNMTs other than the RNA-specific DNMT2 and DNMT6 (Fig. 1A) (*3*, *7*, *37*). Among other holozoans, we could only find a putative DNMT3 in the transcriptome of the choanoflagellate *Achanthoeca spectabilis* (*31*) and both *Pigoraptor* species (*35*), with the same domain architecture lacking additional protein domains as in *Amoebidium* (fig. S1A). Thus, *Amoebidium* and *Pigoraptor* are the only sequenced unicellular holozoan species that retain the ancestral eukaryotic complement of DNMTs, highlighting the pervasive tendency of eukaryotes to lose 5mC.

Next, we performed whole-genome DNA methylation profiling to analyze the 5mC patterns in *Amoebidium*, as well as in three other ichthyosporean species lacking DNA DNMTs as negative controls. In *Amoebidium*, global methylation levels soar to 40%, exclusively within the CG dinucleotide context, setting it apart from most invertebrates and fungi and the other ichthyosporean species, which exhibit negligible levels of 5mC (Fig. 1B) (*1*, *24*). Notably, not all CG dinucleotides exhibit uniform methylation levels. Specifically, the symmetrical mCGC and GmCG trinucleotides stand out with hypermethylation levels at around 70%, whereas the remaining CG dinucleotides maintain lower levels at approximately 20% (fig. S3A). This suggests that *Amoebidium* boasts elevated methylation levels with a wider sequence specificity beyond the CG dinucleotide, a context-dependent regionalization of 5mC reminiscent of heterochromatin methylation in mammals (*38*), likely reflecting the sequence preferences of the diverse *Amoebidium* DNMTs.

Considering the high global methylation levels in *Amoebidium*, we proceeded to investigate which genomic regions exhibit enriched 5mC. Protein-coding genes displayed a gene body methylation pattern reminiscent of plants and animals, with relatively low levels of promoter methylation (Fig. 1C) (*39*, *40*). However, *Amoebidium*’s gene body methylation is not positively correlated with transcription as in plants or animals, as all active genes have similar methylation levels irrespectively of transcriptional level, whereas silent genes show higher methylation, including the promoter (Fig. 1C and fig. S3B). Therefore, gene body methylation appears not exclusive to animals in the holozoan clade, yet its positive association with transcription is an animal-specific feature potentially linked to the domain acquisitions of animal DNMT3s (fig. S1A).

In contrast to most invertebrates (*1*, *2*), *Amoebidium* exhibits targeted methylation of TEs (Fig. 1D and fig. S3, B and C). Notably, methylation levels are highest in recent TE insertions and on transcriptionally silent genes (Fig. 1D and fig. S3C), regardless of the adjacent CG sequence context. In contrast, gene body methylation of actively transcribed genes primarily occurs within the CGC/GCG trinucleotide context (Fig. 1C). This indicates that in *Amoebidium*, 5mC of CGs in non-CGC/GCG trinucleotide context correlates with silencing, whereas CGC/GCG methylation is widespread. Further supporting the link between 5mC and TE silencing, approximately 50% of *Amoebidium*’s genome is composed of TEs, a level unmatched in any unicellular holozoans, yet similar to vertebrates such as humans (50%) or zebrafish (Fig. 1B and fig. S2, C and D). Therefore, the genome of *Amoebidium* is possibly permissive to TE expansions because 5mC can silence these novel insertions by reducing their potential deleterious effects, similar to what has been proposed for vertebrates (*41*),

### Large hypermethylated regions uncover hundreds of viral insertions

**Fig. 2.**
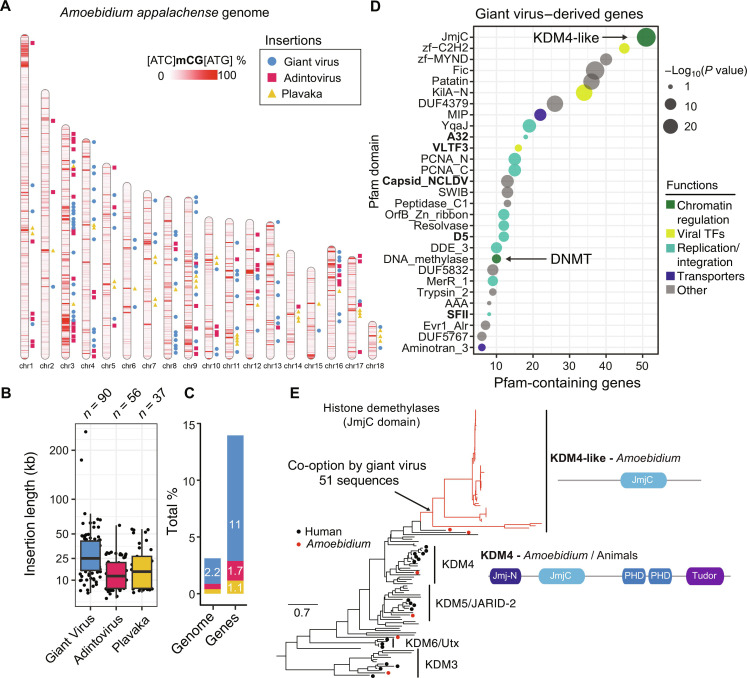
The *Amoebidium* genome harbors hundreds of viral insertions. (**A**) Location of GEVEs, adintovirus, and Plavaka giant repeats across the *Amoebidium* genome. Windows of 10 kb are colored according to their methylation level in non-CGC/GCG trinucleotides. (**B**) Distribution of insertion sizes of the giant repeats within chromosomes. Center lines in boxplots are the median, box is the interquartile range (IQR), and whiskers are the first or third quartile ± 1.5× IQR. (**C**) Contribution of giant repeats to genome size and gene counts. (**D**) Pfam domains enriched in genes encoded in GEVE regions. In bold, marker giant virus domains. The displayed *P* values correspond to a two-sided Fisher exact test. (**E**) Maximum likelihood phylogeny of JmjC in eukaryotes, highlighting the expansion of KDM4-like enzymes in GEVE regions. Black dots indicate human sequences, red dots indicate *Amoebidium* sequences, and red branches indicate genes within endogenized viral regions. Domain architectures defined with PFAM domains.

To characterize the chromosome-level distribution of 5mC, we took advantage of the relative depletion of non-CGC/GCG methylation to locate regions of hypermethylation across the genome. We found many islands of hypermethylation spread across the chromosomes (Fig. 2A), many of which were consistent with regions of high TE content. However, many presented highly gene-rich areas spanning up to 200 kb, with most genes showing few to no introns, in clear contrast to the intron-rich *Amoebidium* genes (average 7.2 exon/gene; fig. S4A). Further characterization of these areas revealed core giant virus genes, including poxvirus late transcription factor (VLTF3), A32-like packaging adenosine triphosphatase (ATPase), D5 DNA primase, or nucleocytoplasmic large DNA viruses (NCLDV) major capsid proteins (fig. S4A) (*14*, *42*). Using these core genes, we searched the National Center for Biotechnology Information (NCBI) database and performed phylogenetic analyses using curated databases of giant virus marker genes (*42*). We found that these insertions could be classified as belonging to a lineage belonging to the order pandoravirales, closely related to the *Mamonoviridae* family of Medusavirus and Clandestinovirus (infecting amoebozoans; fig. S5, A and B) (*43*). Yet, not all the giant endogenous viral elements (GEVEs) in the *Amoebidium* genome originate from a single catastrophic insertion event or even a single viral lineage, as they show high levels of sequence divergence among them (fig. S5C). Furthermore, there are insertions in almost all chromosomes (90 chromosomal insertions, with 42 sequences in unplaced contigs), some having accumulated secondary TE insertions (Fig. 2A). The disparity of insertion lengths, and the observation that none of them encode a full repertoire of core giant virus genes (Fig. 2B), suggests that complete viral genome integrations are rare or that gene loss occurs rapidly after insertion. Using ViralRecall to detect giant viruses solely based on sequence (*44*), we confirmed the presence of GEVEs in *Amoebidium*, yet we failed to recover any hits from other holozoans other than in *Pigoraptor*, which encodes for few giant virus markers, including a capsid protein or a VLTF3, yet these branch far from *Amoebidium* hits (fig. S5, A and B), suggesting that distant classes of giant viruses might endogenize into *Pigoraptor* genomes. However, the fragmented status and metagenomic source of *Pigoraptor* genome assemblies render confident GEVE identification problematic, as some might belong to viruses or other species found in the complex cultures.

In addition to the giant viruses, other compact hypermethylated regions in the *Amoebidium* genome were characterized by genes encoding VLTF3, Dam methyltransferase, minor and major capsid proteins, and a DNA polymerase family B (fig. S4B). DNA polymerase sequences produced closest matches to adintovirus (family *Adintoviridae*), a group of recently described double-stranded DNA polinton-related viruses thought to exclusively infect animals (fig. S5D) (*45*). It is worth noting that many polinton-related viruses are virophages or descendants of these (*46*, *47*), known to parasitize giant viruses, which could explain the abundance of these sequences in the *Amoebidium* genome. Similarly to the giant viruses, not all adintoviruses were closely related among each other (fig. S5E), suggesting multiple independent insertion events. In contrast to GEVEs, some insertions kept long terminal repeats and were complete (~30 kb; Fig. 2B), yet others were truncated and in the process of degeneration.

Then, we identified a third type of giant repeat in *Amoebidium*, consisting of tandem clusters of repetitive intron-poor genes up to 50 kb long, usually flanked by a Plavaka transposase (fig. S4C) (*48*). Many of these genes encode for tyrosine recombinases, one of the major type of transposon integrases in eukaryotes (*49*), and interestingly their only hits in the NCBI NR database belong to very distant eukaryotic lineages including dinoflagellates or red algae, thus suggesting some form of lateral gene transfer as their source (fig. S4C). When we combine the three types of highly methylated giant repeats, they make up 3.1% of *Amoebidium*’s total DNA. Their contribution to the protein-coding genes constitutes 14% of the entire proteome, with the majority originating from viruses. The amount of giant virus insertions in *Amoebidium* is among the largest reported in eukaryotes, at par with the moss *Physcomitrium patens* (Fig. 2C) (*50*).

### Endogenized giant virus co-opted eukaryotic histone demethylases

To understand the potential contribution of endogenized genes to the *Amoebidium* gene repertoire, and also to better understand the gene complement of the original giant virus genomes that infect *Amoebidium*, we characterized the functional enrichment of genes encoded in these endogenized regions. An enrichment test of Pfam domains revealed many domain categories involved in the viral replication and integration process [recombinases, integrases, proliferating cell nuclear antigen (PCNA)], viral gene regulation (transcription factors), or some transporters [e.g., aquaporins/Major Intrinsic Protein (MIP)], which are likely critical to taking control of the host during infection (Fig. 2D) (*51*–*53*). Gene ontologies also suggested that these genes were enriched in membrane fission or tubulin depolymerization (fig. S6A). Notably, some of the most enriched categories were involved in chromatin regulation. Among these, 10 of the 18 DNMTs encoded in the *Amoebidium* genome reside in GEVEs, which suggests that these could be used by the virus to modify its own DNA. Consistently, giant viruses, and members of the pandoravirales in particular, are known to use various forms of DNA methylation (*N*^6^-methyladenine and *N*^4^-methylcytosines) to methylate their own genomes (*54*), which might play a role in infection. However, the *Amoebidium* GEVE DNMTs form a sister group to other giant virus uncharacterized DNMTs (fig. S1B); thus, they were not recently acquired from the host and their sequence-substrate preferences remain unknown.

The most enriched endogenized domain is the Jumonji C (JmjC) domain. Although JmjC domains can perform many enzymatic functions, our phylogenetic analysis revealed that these are divergent paralogs of the histone lysine demethylase subfamily 4 (KDM4). Notably, despite JmjC-containing proteins having been identified in giant viruses (*55*), we could not find any KDM4-like JmjC homologs in publicly available giant virus genomes. *Amoebidium* encodes a canonical KDM4 ortholog like those of other eukaryotes, including its characteristic histone-interacting domains (PHD, Tudor; Fig. 2E). However, the endogenized KDM4-like enzymes only contain the enzymatic JmjC domain (Fig. 2E). KDM4 enzymes are known to demethylate histone 3 tail lysines, most commonly lysine 9 (H3K9me2/3) or lysine 36 (H3K36me2/3) residues. Although many giant viruses encode all four eukaryotic nucleosome histones (H2A/B,H3,H4) (*42*, *56*), we did not find any in the viral insertions. Furthermore, viral histones present very divergent histone tails (*57*); thus, it is unlikely that KDM4-likes are used to control potential giant virus histones. Instead, given the conserved role of H3K9me3 in heterochromatin formation in eukaryotes, KDM4-like enzymes could be used by the virus to avoid silencing by the host chromatin. In KDM4-overexpressing cancer cells, depletion of H3K9me3 promotes DNA breaks and genome instability (*58*), a process that could serve the virus to integrate into the host genome, or explain the amount of endogenization events.

The KDM4-like enzymes stand out among the endogenized genes as they have preserved the multi-exon domain structure of eukaryotic genes (fig. S6C), unlike the vast majority of GEVE genes that lack introns. While most of the endogenized genes remain silent in culture conditions, four of these KDM4-like genes are transcribed [Transcripts Per Million (TPM) > 1] (fig. S6C). Moreover, JmjC genes are found in 39% (*52*) of the insertions, which could reflect a lower chance of purging those genes after the insertion event. A couple of KDM4-like genes are found outside hypermethylated giant virus regions and are flanked by normal host genes, showing almost exclusively mCGC/GmCG methylation (the default state for transcribed host genes; fig. S6C). Given their basal position in the phylogeny of KDM4-likes (Fig. 2E), these genes could be *Amoebidium*-specific KDM4 divergent paralogs that already lost the chromatin interaction domains compared to the canonical KDM4 copy, and were later co-opted by the giant viruses. Alternatively, the giant virus might have originally acquired a canonical KDM4 gene from the host, which then lost some of its companion domains to perform virus-associated functions. Then, these basally branching KDM4-like genes are the remnants of past GEVE insertions, where most other viral genes have been purged and only JmjC loci are kept, being domesticated to become part of the host repertoire. Thus, the intricate interaction between the host chromatin and the giant viruses is likely critical to explain the gene flow between the host and parasite.

### DNA methylation removal is sufficient for viral transcriptional reactivation

**Fig. 3.**
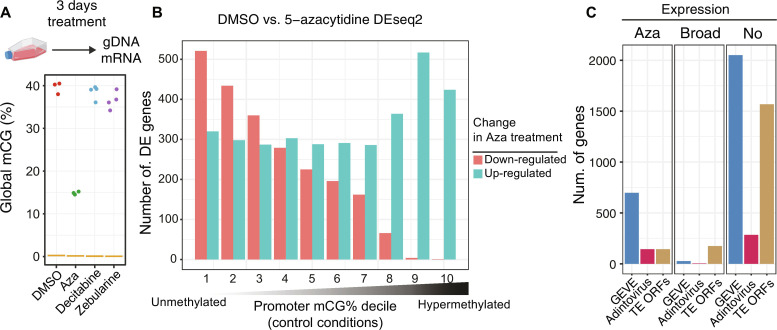
5mC removal leads to viral transcriptional reactivation. (**A**) Global methylation levels measured with Enzymatic Methyl-seq for *Amoebidium* cultures treated for 3 days with DMSO (three biological replicates), 5-azacytidine (Aza; three biological replicates), decitabine (four biological replicates), and zebularine (four biological replicates). (**B**) Distribution of differentially expressed genes classified according to the promoter methylation status in untreated conditions (divided in deciles). The bar color depicts the direction of change upon 5-azacytidine treatment. (**C**) Number of genes encoded in GEVEs, adintoviruses, or TE open reading frames according to their transcriptional response to 5-azacytidine treatment. “Aza” are genes that are only transcribed upon treatment, “Broad” are genes that are expressed in any moment of *Amoebidium* development/control conditions, and “No” are genes that are not expressed in any condition (TPM < 1).

Since dense 5mC demarcates the viral insertions and these are transcriptionally silent, we wanted to directly investigate the causal relationship between 5mC and gene expression in *Amoebidium*. We tested the effect of cytidine analogs 5-azacytidine, zebularine, and decitabine [which block DNMTs and lead to passive dilution of 5mC (*59*, *60*)] to investigate the impact of 5mC on gene expression. A 3-day cytidine analog treatment spans at least two generations of *Amoebidium* colonies, covering two rounds of coenocytic development starting from an uninucleate cell to colony maturation and cell release in ~30 hours (fig. S7A). Therefore, several rounds of nuclear division maximize the potential of obtaining sufficient passive 5mC loss. 5mCG remains constant across development, thus minimizing the potential confounding staging effects across treatments (fig. S7, B and C). We then used Enzymatic Methyl-seq to quantify 5mC of the treated cells and found that only 5-azacytidine showed a decrease in global methylation levels (from ~40 to 15%; Fig. 3A). Consistently, only the *Amoebidium* cells treated with 5-azacytidine showed growth defects and increased mortality (fig. S7D). However, 5-azacytidine can potentially be incorporated into RNA and be cytotoxic (*61*, *62*). To control for those off-target effects, we also treated two ichthyosporean species lacking genomic 5mC with 5-azacytidine, observing mild growth defects (fig. S7E).

We then used RNA sequencing (RNA-seq) to characterize the transcriptional response to 5-azacytidine in *Amoebidium* and *Sphaeroforma arctica*. *Sphaeroforma* is a closely related ichthyosporean that also has a relatively large amount of TEs and few instances of polinton-type viruses (*63*), yet lacks genomic 5mC (Fig. 1B). Both species showed hundreds of differentially expressed genes upon treatment (5630 in *Amoebidium* and 1807 in *Sphaeroforma*; false discovery rate < 0.01), but very few of these showed consistent dynamics across species (fig. S8A), thus not suggesting generic stress response shared across species. Nevertheless, genes that were up-regulated upon 5-azacytidine treatment in *Amoebidium* have stress-associated gene ontologies, while a wide range of metabolic processes are down-regulated (fig. S8D). As observed in 5-azacytidine–treated cancer cells, the stress response might be driven by TE reactivation (*64*). Focusing on TEs, only *Amoebidium* showed a drastic expression increase in almost all TE types after 5-azacytidine (fig. S8B), whereas *Sphaeroforma* did not show any particular enrichment in TE or viral up-regulation (most remaining transcriptionally silent/unchanged; fig. S8B), suggesting that the TE response to 5-azacytidine is a direct consequence of 5mC loss. We further validated this observation by dividing *Amoebidium* genes according to their promoter methylation level in untreated conditions. Genes that normally present unmethylated promoters had a mixed transcriptional response to DNA methylation removal suggestive of indirect effects, whereas genes with hypermethylated promoters were almost exclusively up-regulated upon 5-azacytidine treatment (Fig. 3B). Thus, 5mC is a silencing mark in *Amoebidium* sufficient to repress methylated genes.

When inspecting giant virus and adintovirus endogenized genes, we saw a consistent transcriptional reactivation upon methylation removal. Seven hundred thirty-seven genes encoded in GEVEs (26%) were transcriptionally reactivated (Fig. 3C), with the majority of them being the JmjC genes, but also many genes involved in gene regulation (fig. S8C). Similarly, 144 adintovirus genes were reactivated upon demethylation (32%). However, we did not observe formation of viral particles through microscopy, and consistently, we did not see transcriptional reactivation of capsid proteins. This suggests that viral formation would require extra genes that might have been purged or have accumulated critical mutations since the insertion occurred. Alternatively, posttranscriptional silencing mechanisms could stop the formation of mature viral particles. In sum, direct manipulation of the host methylome demonstrates that 5mC is instrumental for silencing and minimizing the consequence of viral DNA acquisition.

### Giant virus endogenization is polymorphic and highly dynamic in *Amoebidium*

**Fig. 4.**
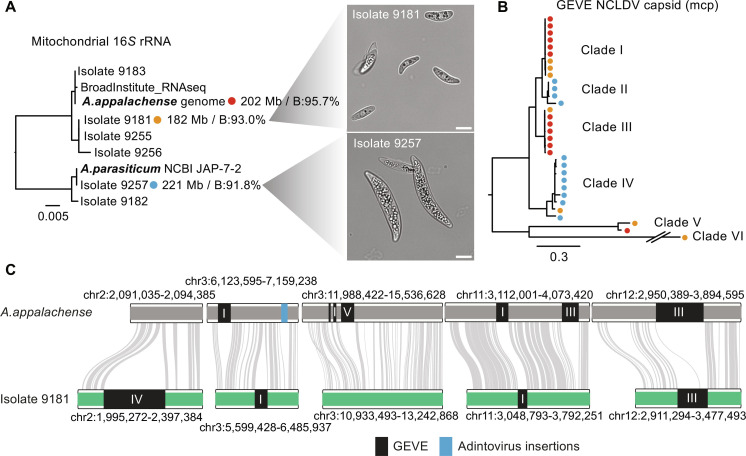
*Amoebidium* isolates display rapid turnover of viral endogenization events. (**A**) Maximum likelihood phylogenetic tree displaying the mitochondrial 16*S* phylogeny of *Amoebidium* isolates. The reference genome (red), isolate 9181 (orange), and isolate 9257 (blue) display the genome assembly characteristics: size (megabases) and BUSCO completeness (B %). Micrographs display *Amoebidium* isolate cells in culture, with the white bar spanning 10 μm. (**B**) Maximum likelihood phylogeny of NCLDV Major capsid proteins encoded in GEVEs. Dots represent the genome they come from following panel (A) color code. Double slash indicates that the branch has been shortened for display purposes. (**C**) Conserved and polymorphic viral insertions across reference genome and isolate 9181. Gray lines indicate the presence of one-to-one orthologs, whereas dark rectangles indicate viral insertions. Roman numerals indicate the clade of the GEVE according to panel (B) phylogeny.

Maintaining a substantial quantity of potentially harmful viral DNA in the *Amoebidium* genome may serve as an adaptive mechanism with important roles. Conversely, it could also represent a passive outcome facilitated by epigenetic silencing. To assess these hypotheses, we set out to compare genetically distinct *Amoebidium* isolates from our reference genome. We first obtained the transcriptome of six isolates, four belonging to *A. appalachense* and two to *A. parasiticum*. Whereas the isolate’s 18*S* sequences were identical at the species level (fig. S9A), the rapidly divergent mitochondrial 16*S* revealed four clades, including a slightly divergent *A. appalachense* lineage (Fig. 4A). We selected a member of the divergent *A. appalachense* lineage (isolate 9181) and one *A. parasiticum* (isolate 9257) for genome sequencing using nanopore long reads. Genome assembly size varied across isolates (Fig. 4A), yet annotation qualities and nanopore-assessed 5mC levels were consistent (fig. S9B).

Annotation of the viral endogenizations in these alternative genotypes revealed a dynamic and diverse history for GEVEs and adintoviruses associated with the *Amoebidium* lineage. Phylogenetic markers such as the major capsid proteins or VLTF3 revealed that at least six separate clades of giant viruses infect these protists, with some clades unique to one isolate (clade II) and others shared by the isolates (clade IV) but absent in the reference genome (Fig. 4B and fig. S9C). Similarly, four adintovirus clades are found across the isolates, with some being shared across all three genomes (fig. S9D). Notably, isolate 9257 shows only 4 adintoviruses compared to the 44 present in the reference genome. This reveals that viral diversity infecting *Amoebidium* is not limited to a single lineage and is often endogenized in an isolate-specific manner.

Since sequence similarity and gene synteny between isolate 9181 and the reference genome remains highly conserved (fig. S9E), we used this to assess the ancestral nature of endogenization events. Despite their close phylogenetic relationship, only a minority of endogenization events were shared across the isolates, with most featuring polymorphic insertions amidst synteny blocks (Fig. 4C). Giant viruses are not known to require integration into the host genome during their infectious cycles (*14*); this process appears to be stochastic, potentially occurring during unsuccessful infections and at various chromosome positions. Additionally, it underscores the dynamic nature of integration, which the host tolerates through multiple cycles, with most endogenized elements being quickly eliminated after insertion.

## DISCUSSION

**Fig. 5.**
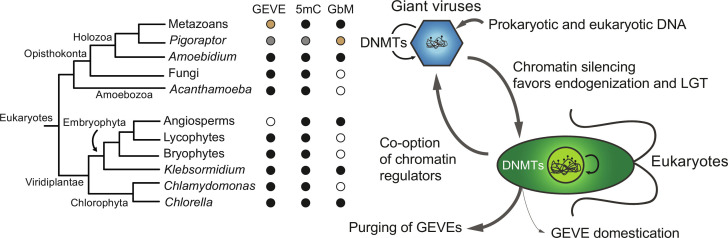
Giant virus endogenization events correlate with the presence of 5mC in eukaryotes. Cladogram representing the lineages where large GEVEs have been described in the literature, and for which 5mC and gene body methylation pattern (GbM) presence has been reported. Dark dots indicate presence, white dots indicate absence, gray dots indicate that it is highly likely (due to the presence of DNMT1/3), and brown dots indicate lack of data/studies. Schematic of the lateral gene transfers (LGT) from giant viruses to eukaryotes, and vice versa, mediated by chromatin regulatory mechanisms. DNMTs mediate DNA methyl marks shown as lollipops.

Here, we show how a unicellular eukaryote closely related to animals undergoes a recurrent process of mixing its genome with that of its giant virus predators. This foreign DNA is curbed by 5mC silencing, allowing for survival after these potentially lethal events. We propose that epigenetic silencing greatly reduces the lethality of these endogenization events. Supporting this general hypothesis, many of the previously described large-scale giant virus endogenizations in eukaryotes, including early land plant lineages, the fungus *Rhizophagus irregularis*, green algae, or the amoebozoan *Acanthamoeba castellanii*, coincide with species that have retained 5mC as a silencing mechanism (Fig. 5) (*19*–*21*, *65*, *66*). Plants that are not dependent on water for reproduction (Spermatophyta) or germline-segregating animals are likely protected from giant virus endogenization events despite carrying silencing mechanisms (*20*), yet chromosome-scale genomes and directed searches might reveal exceptions to this rule. It is also possible that some eukaryotic lifestyles might make some species less likely to be infected by giant viruses, such as that of internal parasites, or that extreme genome compaction requirements make endogenizations unlikely to be fixed in a population, such as in prasinophytes. Giant viruses infect all kinds of eukaryotic groups, including species with and without DNMTs. However, eukaryotes that have secondarily lost 5mC silencing, as exemplified by most of the available unicellular holozoan genomes, rarely present large giant viral DNA insertions. Notably, most eukaryotic clades have genes derived from giant viruses (*13*), or insertions of double-stranded medium size DNA viruses like polinton-like/virophages (*63*), suggesting that infection and endogenization are widespread, but the retention of these insertions is uneven across lineages. It is likely that other silencing mechanisms other than 5mC, such as histone modifications (e.g., H3K27me3 or H3K9me3) or small interfering RNAs (siRNAs), can be used for silencing GEVEs, as exemplified by H3K79me2 in the brown algae *Ectocarpus siliculosus* GEVE (*67*–*69*), a species that lacks DNMTs and 5mC. Yet, possibly the cost of integrating large amounts of viral DNA in epigenetically unprotected species is coped with by extremely rapid purging or takeover by uninfected conspecific cells.

The 5mC patterns in *Amoebidium* also suggest that gene body methylation predates animal origins. Although this would potentially support the hypothesis that gene body methylation was present in the ancestor of eukaryotes (*39*), this pattern is very sparsely distributed (Fig. 5). Among chlorophytes, only *Chlorella variabilis* has a pattern similar to that of *Amoebidium* (*39*), while absent in *Chlamydomonas* and Prasinophytes (*5*, *40*). Spermatophytes [angiosperms (*70*), conifers (*71*), and ferns (*72*)] show gene body methylation, whereas liverworts and mosses generally lack it (*39*, *73*, *74*). In contrast, the more basally branching streptophyte *Klebsormidium nitens* shows gene body methylation (*65*), albeit in a pattern quite divergent to land plants, which could suggest independent origins of gene body methylation in these lineages. *Amoebidium*, *Chlorella* (*75*), and *Klebsormidium* (*65*) present giant virus endogenization events, which suggests that gene body methylation might arise as a convergent response or by-product to recurrent infections and expansion of parasitic DNA (*70*), perhaps avoiding intra-genic elements hijacking transcription from the host genes (*76*). In particular, *Amoebidium* encodes DNMT3 and an animal-like DNMT1, which could support that gene body methylation across holozoans is homologous and deposited by orthologous DNMTs. In the future, if 5mC data can be obtained from *Pigoraptor* species, it will help to elucidate if *Amoebidium* represents a case of convergent evolution of gene body methylation or this is ancestral to holozoans. With available data, the link with gene body and transcription appears an animal innovation, enabled through the acquisition of PWWP and ADD domains in animal DNMT3 orthologs, starting a feedback loop with histone modifications such as H3K36me2/3 (*77*–*79*). Regulation of host gene transcription in multicellular animals might have restricted and weakened the role of 5mC in TE silencing, suggested by its absence across many invertebrate genomes (*1*, *2*).

Giant viruses emerged before the origins of modern eukaryotes (*16*), and chromatin silencing mechanisms such as 5mC or histone modifications were present in the Last Eukaryotic Common Ancestor (*1*, *9*, *55*). Thus, these patterns of frequent giant virus endogenization that we observe in modern eukaryotes must have been constant during the whole history of the lineage. Although domestication of giant virus–derived genes might be rare, we can see examples of this occurring throughout the tree of life (*13*, *80*, *81*). It is worth highlighting that despite giant virus–derived genes being widespread, their domestication potential is harder to assess, given the difficulty to test their roles and expression across divergent protist species. Thus, giant viruses, whose genetic material is itself a composite of various origins (*82*–*84*), serve as a source of genetic novelty via lateral gene transfer across eukaryotes (Fig. 5). Unlike plasmids or other forms of bacterial lateral gene transfer mechanisms, giant viruses are a dangerous vessel for genetic interchange; thus, chromatin silencing mechanisms are probably required for a stepwise acquisition of foreign DNA. In turn, the host chromatin protection is likely counteracted by giant viruses, as exemplified by the histone demethylases present in *Amoebidium* GEVEs, or other examples of chromatin modifiers reported in giant virus genomes (*55*). Similarly, the presence of DNMTs in GEVEs, and the capacity of giant viruses to modify their own DNA (*54*), could be a protective response against eukaryotic chromatin, avoiding viral DNA to be recognized as a threat. Chromatin hijacking by giant viruses is a process reminiscent of cases in which TEs have co-opted host chromatin regulators (*48*, *65*, *85*, *86*), highlighting the age-long conflict between eukaryotic chromatin and parasitic DNA. In summary, *Amoebidium* exemplifies the intricate network-like origins of eukaryotic DNA, challenging traditional notions of strict vertical inheritance within the clade.

## MATERIALS AND METHODS

### Cell culture, treatment, and nucleic acid extraction

*Amoebidium* isolates were grown on Brain Heart Infusion (10% BHI, Thermo Fisher Scientific CM1135) liquid medium at 25°C in 25-ml culture flasks. *S. arctica, Creolimax fragrantissima*, and *Chromosphaera perkinsii* were grown in liquid Marine Broth (Difco Marine Broth 2216) at 17°C. Six *Amoebidium* alternative isolates were obtained from the ARS Collection of Entomopathogenic Fungal Cultures.

DNA methylation drugs 5-azacytidine (ab142744), decitabine (ab120842), and zebularine (ab141264) were dissolved in dimethyl sulfoxide (DMSO). *A. appalachense* was grown with 0 M, 0.1 μM, 1 μM, 10 μM, 100 μM, and 1 mM final concentration of each drug in 2 ml of 10% BHI with 10% DMSO in a 12-well plate, and effects were tracked daily for 5 days. Only 100 μM and 1 mM 5-azacytidine showed a growth phenotype. *A. appalachense* DNA and RNA were extracted from cultures grown for 3 days in 10 ml of 10% BHI with 1% DMSO, and 1% DMSO with 100 μM of their respective drug, in triplicate. 5-Azacytidine (12 nmol) in 120 μl of DMSO was spread over 12-ml agar plates of BHI (*A. appalachense*) and Marine Broth (*S. arctica*, *C. fragrantissima*, *C. perkinsii*), and dilution assays for growth were done for all four ichthyosporean species using 1×, 10×, 100×, 1000×, and 10,000× serial dilutions of saturated culture.

The developmental cell cycle of *A. appalachense* was determined using a combination of live and fixed-cell microscopy using a fully motorized Nikon Ti2-E epifluorescence inverted microscope equipped with a hardware autofocus PFS4 system, a Lumencor SOLA SMII illumination system, and a Hamamatsu ORCA-spark Digital CMOS camera. CFI Plan Fluor 20×, 0.50 NA (numerical aperture), CFI Plan Fluor 40× Air, and CFI Plan Fluor 60× Oil, 0.5 to 1.25 NA objectives were used for imaging. For live-cell microscopy, a 25-day-old culture was diluted 1:250 and imaged with bright field every 15 min for 72 hours at a controlled temperature of 23°C in 600-μl wells using a cooling/heating P Lab-Tek S1 insert (Pecon GmbH) with Lauda Loop 100 circulating water bath. We examined 120 videos counting events of spontaneous cell death, cellularization, and cell release, and the number of released spores per colony (total 703 cells tracked; table S1). For fluorescent microscopy, samples were fixed in 4% formaldehyde, washed with phosphate-buffered saline (PBS), and stained with phalloidin and Hoechst to visualize and count actin and nuclei, respectively, every 4 to 5 hours over 72 hours. RNA and DNA were obtained for representative stages of the life cycle: 5 hours after inoculation (unicell—uninucleated), 14 hours (coenocyte), 20 hours (cellularization), 33 hours (cell release).

DNA for *A. appalachense* genome sequencing was extracted using liquid nitrogen grinding and Qiagen MagAttract HMW DNA Kit & QIAGEN Genomic-tip 20/G (10223), and for *A. appalachense*, *S. arctica*, *C. perkinsii*, and *C. fragrantissima* Enzymatic Methyl-seq samples, we used NEB Monarch Genomic DNA Purification Kit. DNA for *Amoebidium* isolates 9181 and 9257 was extracted with phenol chloroform extraction and further purification with NEB Monarch Genomic DNA Purification Kit. RNA for all samples was extracted using nitrogen grinding and Monarch Total RNA Miniprep Kit.

### Micro-C library preparation

*A. appalachense* cells grown for 7 days were crosslinked for 10 min with 1% formaldehyde under vacuum conditions in a desiccator. The reaction was quenched with 128 mM glycine for 5 min under vacuum, followed by an additional incubation on ice for 15 min. Crosslinked cells were washed twice and subsequently resuspended in a ^1^/_10_ PBS solution. Coenocytic cell walls were disrupted by glass bead beating for 5 min followed by a second crosslinking step with 3 mM DSG (disuccinimidyl glutarate) for 40 min at room temperature.

Micro-C libraries were prepared as described (*87*) with the following modifications. In-nuclei chromatin digestion to achieve 80% monomer/20% oligomer nucleosome ratio was performed with 100 U of MNase (Takara Bio, 2910a) per 4 M nuclei for 10 min. The digested chromatin ends were repaired and labeled with biotinylated nucleotides. Before proximity ligation, the digested chromatin was released from nuclei and permeabilized coenocytes by glass bead beating for 10 min. Next, proximal nucleosomes were ligated together, and unligated ends were treated with Exonuclease III (NEB, M0206) to remove biotin-dNTPs (Deoxynucleotide Triphosphates). The chromatin was then decrosslinked and deproteinized, and ligated DNA fragments were captured with Dynabeads MyOne Streptavidin (Life Technologies, 65602). Libraries were barcoded using the NEBNext End repair/dA-tailing mix (NEB, E7546) and NEBNext Ultra II Ligation Module (NEB, E7595S). The final amplified libraries, comprising three biological replicates, were sequenced with NextSeq500 in paired-end format with a read length 42 bases per mate, obtaining a total of 131,547,803 sequenced reads.

### Genome sequencing and assembly

High molecular weight genomic DNA from *A. appalachense* was ligated with the Nanopore SQK-LSK110 ligation kit and sequenced in Promethion R9 flowcells. Since pore clogging occurred quickly, we performed short sequencing runs followed by flowcell cleanup steps, and reloading of fresh library in intervals, requiring three flowcells. In parallel, a library of paired-end short reads was generated with the TruSeq kit and sequenced with an Illumina HiSeq2500. Nanopore reads were basecalled using the “sup” model with Guppy (v6.2.1) and assembled with Flye (v2.9-b1768) with the “--nanopore_hq” parameter and two rounds of polishing (*88*). The resulting genome was further polished with the short reads with Pilon (*89*) for two rounds, using BUSCO score (-m genome, v5) (*90*) to validate improvements, obtaining a contig level N50 of 1.8 Mb. Micro-C data were mapped on the genome using Juicer (v1.6) (*91*) with the -p assembly option. The 3D-DNA pipeline (*92*), using the proximity ligation data, was used to scaffold the genome with -r3 -editor-repeat-coverage 10. Final manual curation in the Juicebox Assembly Tool (*93*) resulted in 18 chromosomes. The genome was then polished using Medaka with the original nanopore reads.

For the isolates 9181 and 9257, we ligated the DNA using Nanopore SQK-LSK114 ligation kit and sequenced following the same strategy but using PromethIon R10 flowcells (table S2). Contig-level assembly was obtained using Flye with Guppy “sup” base called reads as above. Medaka polishing was discarded as it decreased BUSCO score. Then, D-GENIES was used to visualize the synteny with the reference genome (*94*). RagTag scaffolding using the reference genome was performed for both isolate contigs (*95*), yet only 9181 was kept as 98% of the sequence were placed into chromosomes, whereas 9257 only got 54%, rendering the scaffolding unreliable. Extra scaffolding using P_RNA_scaffolder (*96*) was performed for 9257 using its transcriptomic data to further increase contiguity, and validated through BUSCO improvement criteria.

### Genome annotation

We generated a de novo RepeatModeler2 (*97*) annotation with the LTR module to characterize *A. appalachense* repeat landscape. This was then mapped to the genome using RepeatMasker. In parallel, publicly available deep coverage RNA-seq from *A. appalachense* (SRR545192) was mapped to the genome using HISAT2 with the–dta parameter, and Stringtie for reference based transcriptome assembly (*98*). The resulting bam was processed with Portcullis to generate a list of high-quality intron junctions (*99*). In parallel, de novo Trinity assembly of the SRR545192 reads was mapped using gmap to the genome (*100*). The combination of introns, Stringtie, and Trinity mappings was fed to Mikado to choose the best collection of transcripts based on the UniProt Sprot database. The best transcripts were used to train Augustus model for *Amoebidium* (*101*). To inform Augustus annotation, we mapped protein alignments against the genome using MetaEuk (*102*), using closely related high-quality ichthyosporean genomes as query, obtaining coding sequence hints. Portcullis introns and Mikado exons were also introduced as hints for Augustus genome annotation. The resulting Augustus annotation was then updated using PASA with the Mikado transcripts, fixing broken gene models and adding untranslated regions. Annotation was visually inspected in the IGV genome browser. Functional annotation was obtained using hmmscan with Pfam-A database (*103*) and the eggNOG-mapper server (*104*).

To annotate the alternative isolate genomes, the same process was followed, using the reference annotation for the MetaEuk CDS hints, and the pre-trained *Amoebidium* Augustus model. All annotations were evaluated using BUSCO v5 with eukaryota_odb10 database.

### Transcriptome sequencing, assembly, and analysis

We used 50 to 1000 ng of RNA from treated samples, developmental time points, and isolates to build mRNA-seq libraries (see table S3 for details), first enriching for poly-A transcripts with the NEB Magnetic mRNA Isolation Kit S1550S, and then building the libraries with the NEBNext Ultra II Directional RNA Library Prep Kit for Illumina (E7760L) according to manufacturer’s instructions. Short-read Illumina reads were obtained with NovaSeq6000. De novo transcriptome assemblies were obtained with Trinity (strand-specific) for the isolates. The Trinity assemblies were searched for 18*S* and 16*S* sequences using BLASTn with NCBI query sequences.

Drug treatment and developmental samples were mapped against the annotation using Kallisto to obtain TPMs (*105*). To perform differential expression analysis of TEs and protein-coding genes, we used HISAT2 with the TElocal pipeline (*106*), obtaining gene counts that were then analyzed in DEseq2 (*107*). Only intergenic TEs above 500 base pairs (bp) were kept for the analysis. *Sphaeroforma* treatment samples were done in the same way and mapped to the latest version of the genome (*108*).

### Methylome sequencing and analysis

We sonicated genomic DNA from *A. appalachense* (control, developmental time points, DMSO/5-azacytidine treated), *S. arctica, C. perkinsii,* and *C. fragrantissima*, spiked with phage lambda DNA and methylated pUC19 controls, to obtain 300-bp fragments with Covaris M220. Then, we used the NEB Enzymatic Methyl-Kit to convert all the unmethylated Cs into Ts as described in the manufacturer’s instructions (*109*). These libraries were then sequenced in Illumina NovaSeq6000 to various coverages (table S4). The reads were then mapped with fastp and mapped to the reference genomes (*29*, *108*, *110*) using BS-Seeker2 backed with bowtie2 (*111*). Sambamba was used to remove polymerase chain reaction (PCR) duplicates, and CGmapTools was used to obtain the methyl calls (*112*). These files were processed in R using the bsseq package, and bigwig tracks using the BedGraphToBigWig UCSC utility.

In parallel, nanopore reads were basecalled and mapped for base modifications using the Guppy dna_r9.4.1_450bps_modbases_5mc_cg_sup_prom.cfg and dna_r10.4.1_e8.2_400bps_modbases_5mc_cg_sup_prom.cfg models. The resulting read alignments were processed with modbam2bed the --cpg -e -m 5mC parameters. These bed files were also processed in R using the bsseq package.

### Giant virus identification and phylogenetic analysis

Visual inspection of hypermethylated blocks revealed core giant virus genes in unusual gene architecture patterns. To validate these potential claims, we used ViralRecall (*44*) that flagged just a few of these sequences as potential giant virus endogenization events. However, we observed that many events were not captured by that software, so we manually inspected the genome to obtain the longest potential inserts, filtering out TEs inserted within the viral region. We searched those consensus sequences against the genome using BLASTn to obtain all potential regions of homology to giant viruses. Another round of manual inspection of all chromosomes using non-CGC/GCG methylation blocks as boundary demarcation was used to delimit integration sites. The same process was used for adintoviruses and Plavaka giant repeats. We ran ViralRecall on the genomes of other ichthyosporeans (*C. fragrantissima*, *S. arctica*, *Ichthyophonus hoferi*, *C. perkinsii*, *Abeoforma whisleri*, *Pirum gemmata*) (*29*, *110*), *Corallochytrium limacisporum*, the filasterean *Capsaspora owczarzaki* (*113*), and the choanoflagellates *Monosiga brevicollis* and *Salpingoeca rosetta* (*114*, *115*), and we did not obtain any reliable hit on this collection of holozoan genomes (table S5).

Hmmsearch was used to identify core viral genes, DNMTs (PF00145), and JmjC (PF02373)–containing proteins. DNMTs and core NCLDV genes were searched in a large collection of holozoan genomes and transcriptomes, including 22 choanoflagellates (*31*, *116*), 4 filastereans (*35*), 7 ichthyosporeans, and *C. limacisporum* (see table S5). The obtained genes were included to sequences from reference databases (*42*, *45*, *55*) and aligned using MAFFT in lins-i mode (*117*). Alignments were trimmed using TrimAL with the -gappyout mode (*118*). The resulting alignments were fed into IQ-TREE 2 with automatic model testing to build maximum likelihood phylogenetic trees using altr and uboot as nodal support measures (*119*). Adintovirus minor and major capsid proteins were annotated with HHpred against PDB_mmCIF70 database.

Comparative genomics among giant virus insertions (used as independent taxa) or across the isolate genomes was performed using OrthoFinder with DIAMOND as a search engine (*120*).
