# Supplementary material for: DNA methylation enables recurrent endogenization of giant viruses in an animal relative
Source: Sci Adv. 2024 Jul 12;10(28):eado6406. doi: 10.1126/sciadv.ado6406 (PMC11244446; doi:10.1126/sciadv.ado6406)
Supplement: Supplementary file 1 — Figs. S1 to S9 Legends for tables S1 to S5 [file sciadv.ado6406_sm.pdf]

Science Advances
AAAS

Supplementary Materials for

DNA methylation enables recurrent endogenization of giant viruses in an animal relative

Luke A. Sarre *et al.*

Corresponding author: Alex de Mendoza, a.demendozasoler@qmul.ac.uk

Sci. Adv. **10**, eado6406 (2024)
DOI: 10.1126/sciadv.ado6406

The PDF file includes:

Figs. S1 to S9
Legends for tables S1 to S5

Other Supplementary Material for this manuscript includes the following:

Tables S1 to S5

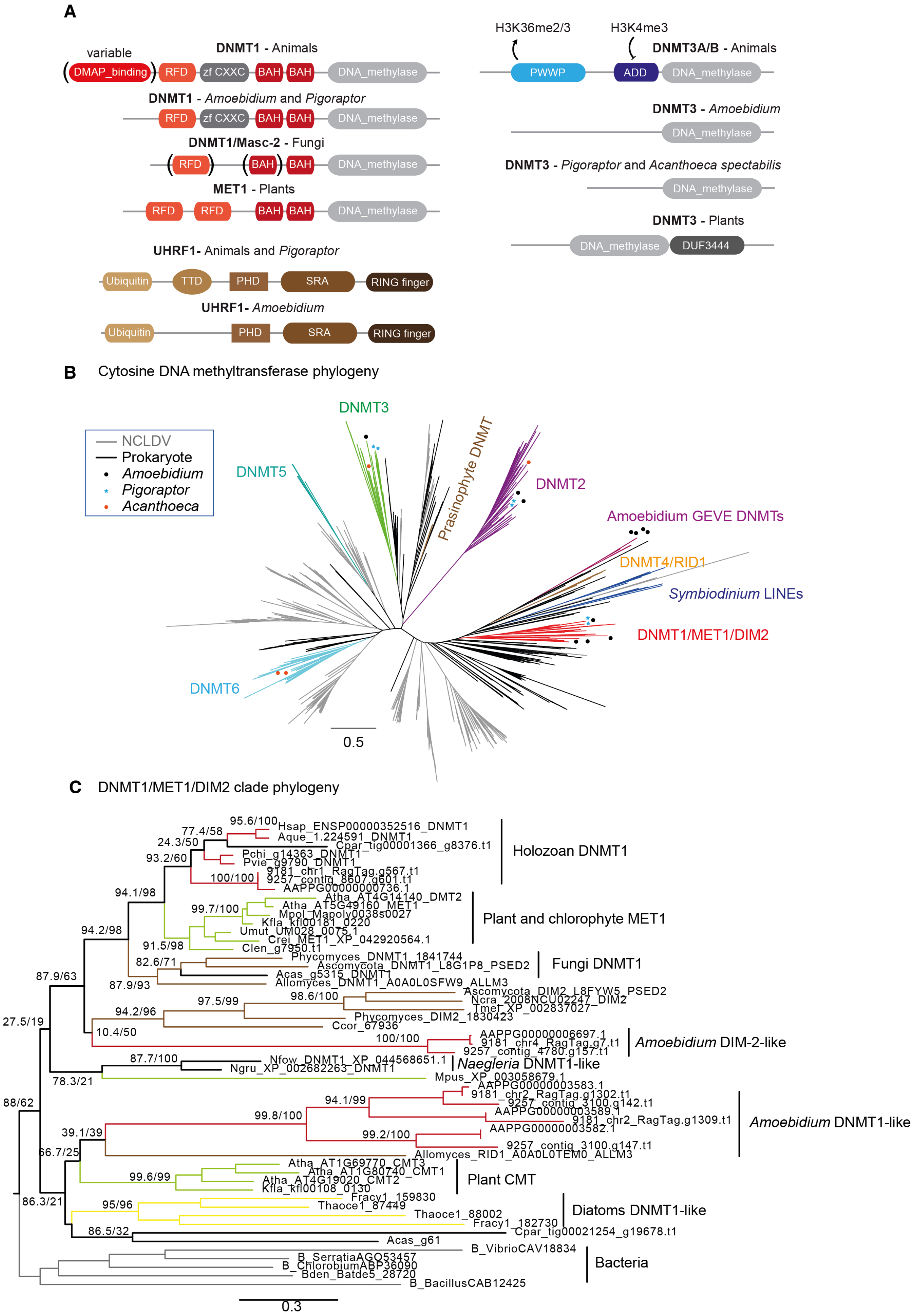

A
variable
DMAP_binding
RFD
zf CXXC
BAH
BAH
DNA_methylase
DNMT1 - Animals
DNMT1 - Amoebidium and Pigoraptor
DNMT1/Masc-2 - Fungi
MET1 - Plants
UHRF1- Animals and Pigoraptor
Ubiquitin
TTD
PHD
SRA
RING finger
UHRF1- Amoebidium
H3K36me2/3
H3K4me3
DNMT3A/B - Animals
PWWP
ADD
DNMT3 - Amoebidium
DNMT3 - Pigoraptor and Acanthoeca spectabilis
DNMT3 - Plants
DUF3444
B Cytosine DNA methyltransferase phylogeny
NCLDV
Prokaryote
Amoebidium
Pigoraptor
Acanthoeca
DNMT3
DNMT5
Prasinophyte DNMT
DNMT2
Amoebidium GEVE DNMTs
DNMT4/RID1
Symbiodinium LINEs
DNMT1/MET1/DIM2
DNMT6
0.5
C DNMT1/MET1/DIM2 clade phylogeny
95.6/100
Hsap_ENSP00000352516_DNMT1
77.4/58
Aque_1.224591_DNMT1
24.3/50
Cpar_tig00001366_g8376.t1
93.2/60
Pchi_g14363_DNMT1
Pvie_g9790_DNMT1
9181_chr1_RagTag.g567.t1
100/100
9257_contig_8607.g601.t1
AAPPG00000000736.1
Holozoan DNMT1
94.1/98
Atha_AT4G14140_DMT2
99.7/100
Atha_AT5G49160_MET1
Mpol_Mapoly0038s0027
Kfla_kfl00181_0220
94.2/98
Umut_UM028_0075.1
Crei_MET1_XP_042920564.1
91.5/98
Clen_g7950.t1
Plant and chlorophyte MET1
82.6/71
Phycomyces_DNMT1_1841744
Ascomycota_DNMT1_L8G1P8_PSED2
Fungi DNMT1
87.9/63
87.9/93
Acas_g5315_DNMT1
Allomyces_DNMT1_A0A0L0SFW9_ALLM3
Ascomycota_DIM2_L8FYW5_PSED2
98.6/100
97.5/99
Ncra_2008NCU02247_DIM2
Tmel_XP_002837027
94.2/96
Phycomyces_DIM2_1830423
27.5/19
Ccor_67936
10.4/50
AAPPG00000006697.1
100/100
9181_chr4_RagTag.g7.t1
9257_contig_4780.g157.t1
Amoebidium DIM-2-like
87.7/100
Nfow_DNMT1_XP_044568651.1
Ngru_XP_002682263_DNMT1
Naegleria DNMT1-like
78.3/21
Mpus_XP_003058679.1
88/62
AAPPG00000003583.1
9181_chr2_RagTag.g1302.t1
94.1/99
9257_contig_3100.g142.t1
99.8/100
AAPPG00000003589.1
9181_chr2_RagTag.g1309.t1
39.1/39
99.2/100
AAPPG00000003582.1
Amoebidium DNMT1-like
66.7/25
9257_contig_3100.g147.t1
Allomyces_RID1_A0A0L0TEM0_ALLM3
Atha_AT1G69770_CMT3
Atha_AT1G80740_CMT1
99.6/99
Atha_AT4G19020_CMT2
Kfla_kfl00108_0130
Plant CMT
86.3/21
95/96
Fracy1_159830
Thaoce1_87449
Diatoms DNMT1-like
Thaoce1_88002
Fracy1_182730
86.5/32
Cpar_tig00021254_g19678.t1
Acas_g61
B_VibrioCAV18834
B_SerratiaAGO53457
B_ChlorobiumABP36090
Bden_Batde5_28720
B_BacillusCAB12425
Bacteria
0.3

Fig. S1.

Distribution of DNMTs in *Amoebidium*. (**A**) Protein domain architectures of DNMT members of DNMT1 and DNMT3 families as well as the UHRF1, the heterodimer of DNMT1, in various eukaryotic groups. Domains are defined by PFAM: DMAP_binding (PF06464), RFD (PF12047), zf-CXXC (PF02008), BAH (PF01426), DNA_methylase (PF00145), Ubiquitin (PF00240), Tandem Tudor Domain (TDD, PF12148), PHD (PF00628), SRA (PF02182), PWWP (PF00855), ADD (PF17980). Parenthesis indicates variability in the presence of the domain within the group. Arrows indicate that the PWWP domain of DNMT3A/B orthologues in mammals binds to H3K36me2/3, whereas the ADD domain prevents binding to H3K4me3 marked regions. (**B**) Maximum-likelihood phylogenetic tree of DNMTs including prokaryotic, eukaryotic and giant virus sequences. Known eukaryotic groups are depicted with colours, and presence of *Amoebidium* sequences are highlighted with a black dot. (**C**) Maximum-likelihood phylogeny of the clade of DNMT1 and DNMT1-like sequences in eukaryotes, rooted with the closest bacterial sequences. Nodal supports display ALRT and uboot values calculated by IQTREE. The bottom bar indicates substitutions per site. Species IDs: Atha (*Arabidopsis thaliana*), Mpol (*Marchantia polymorpha*), Kfla (*Klebsormidium nitens*), Crei (*Chlamydomonas reinhardtii*), Umut (*Ulva mutabilis*), Clen (*Caulerpa lentillifera*), Hsap (*Homo sapiens*), Aque (*Amphimedon queenslandica*), Cpar (*Cyanophora paradoxa*), Acas (*Acanthamoeba castellannii*), Ncra (*Neurospora crassa*),Tmel (*Tuber melanosporum*), Ccor (*Conidiobolus coronatus)*, Fracy (*Fragilariopsis cylindrus*), Thaoce (*Thalassiosira oceanica*), Mpus (*Micromonas pusilla*), Ngru (*Naegleria gruberi*), Nfow (*Naegleria fowleri*), AAPPG/9181/9257 (*Amoeibidium* and isolates), Pchi (*Pigoraptor chileana*), Pvie (*Pigoraptor vietnamica*).

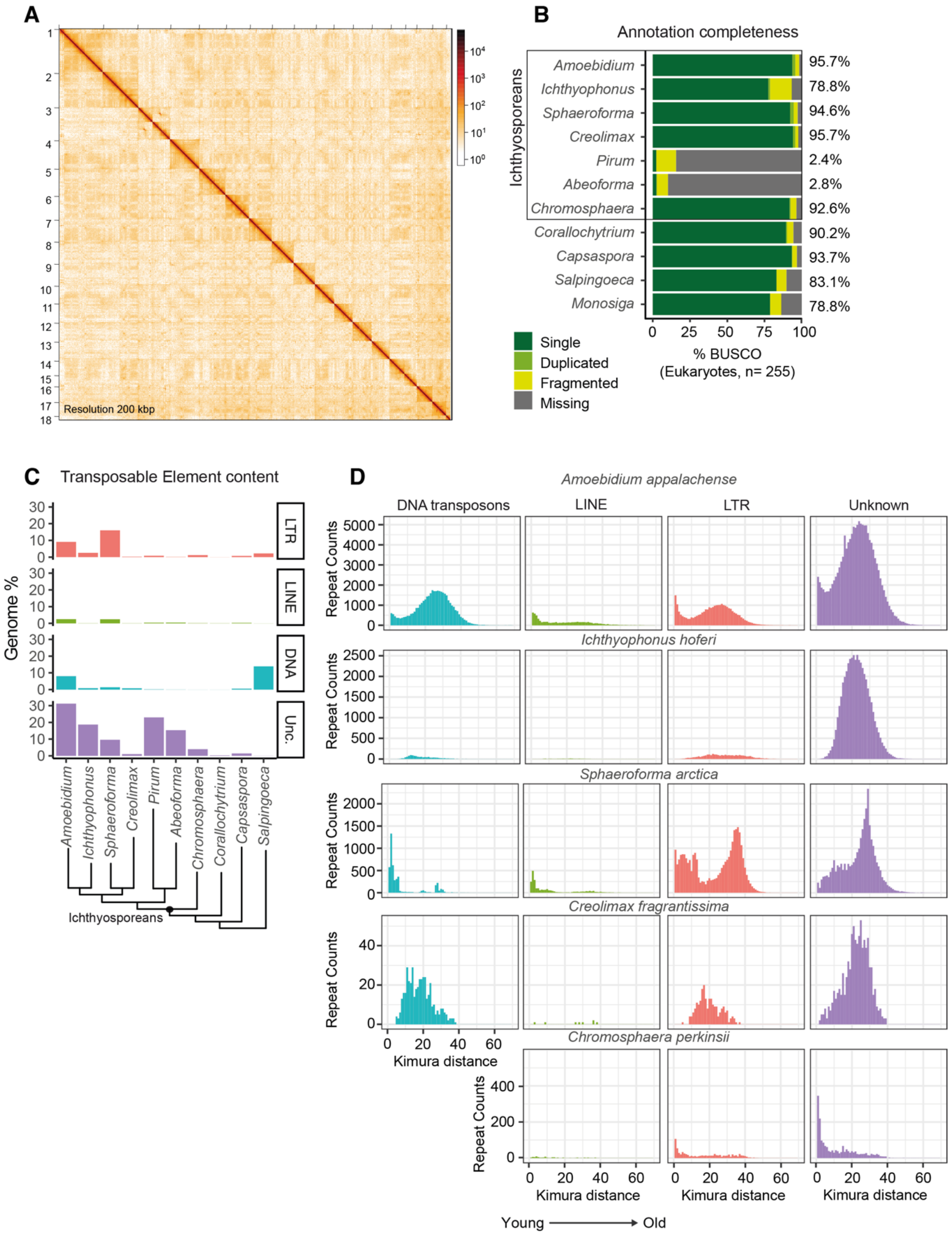

Fig. S2.

Genome assembly, completeness and repeat landscape in *Amoebidium*. (**A**) Heatmap representing the micro-C contact map across and within *Amoebidium* chromosomes, with a

window resolution size of 200 kb. (**B**) BUSCO comparison across published unicellular holozoan genomes, with the total BUSCO % (Single + Duplicated %) displayed on the right hand side. (**C**) Major TE type contributions to the genomes of selected unicellular holozoans. TEs annotations have been obtained with RepeatModeler2 and RepeatMasker. LTR stands for long terminal repeat retrotransposons, LINE for non-LTR retrotransposons, DNA for DNA transposons and Unc for Unknown / unclassified repeats. (**D**) Age profile of repeats across high quality ichthyosporean genomes obtained as Kimura distances (not corrected for CpGs).

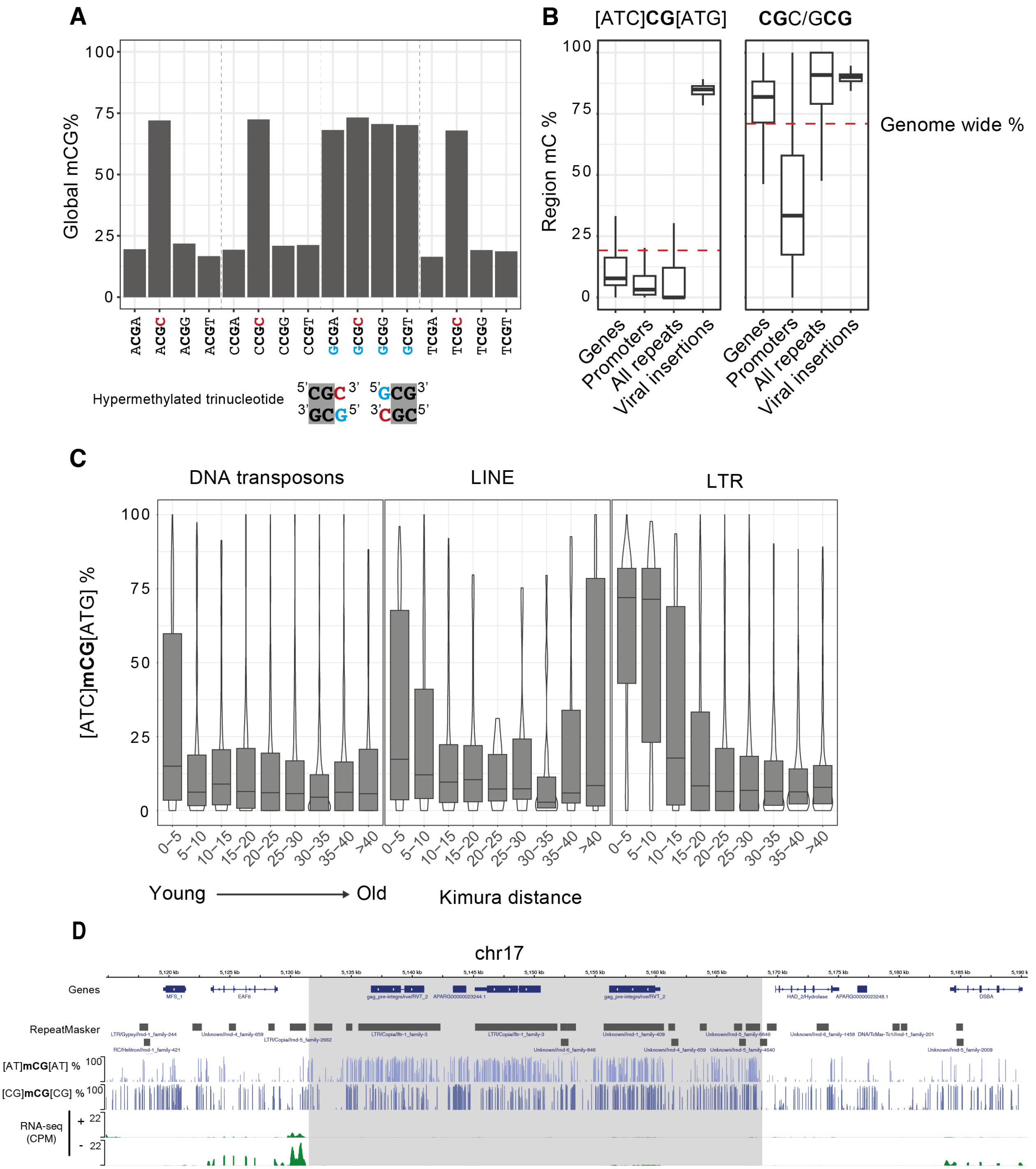

Fig. S3.

Trinucleotide context influences CG methylation distribution in *Amoebidium*. (**A**) Genome wide methylation levels on CG dinucleotides surrounded by all possible base compositions measured by Enzymatic Methyl-seq. (**B**) Regional methylation across genes, promoters, repeats and viral insertions divided by trinucleotide context. (**C**) non-CGC/GCG methylation distribution on TEs classified by age. TE insertions with Kimura distances spanning 5% differences are shown from left to right. Centre lines in boxplots are the median, box is the interquartile range

(IQR), and whiskers are the first or third quartile ± 1.5× IQR, outliers not shown. (**D**) Genome browser snapshot of retrotransposons methylated in all CG contexts, and neighbouring genes predominantly displaying CGC/GCG methylation.

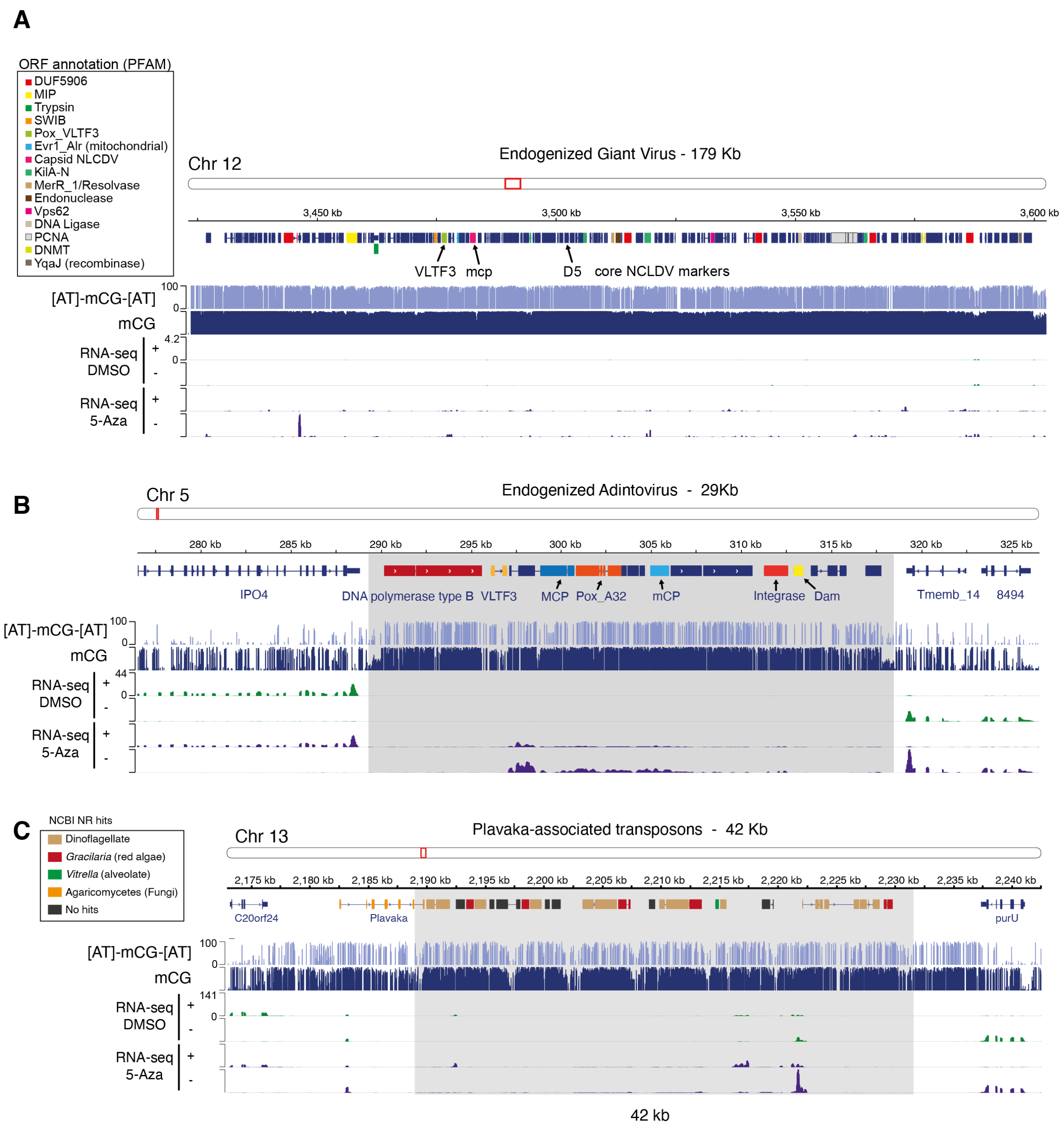

Fig. S4.

The three major types of giant repeats in the *Amoebidium* genome. (**A**) Genome browser snapshot of a large GEVE in chromosome 12. Coloured genes highlight various PFAM containing Open Reading Frames. (**B**) Genome snapshot of an Adintovirus insertion spanning 29 kb, flanked by host protein coding genes. mCP: minor capsid protein, MCP: major capsid protein. (**C**) Plavaka-associated giant repeat spanning 42 kb. Genes are colour coded according to the taxonomy of the first hits against the NCBI non-redundant protein database.

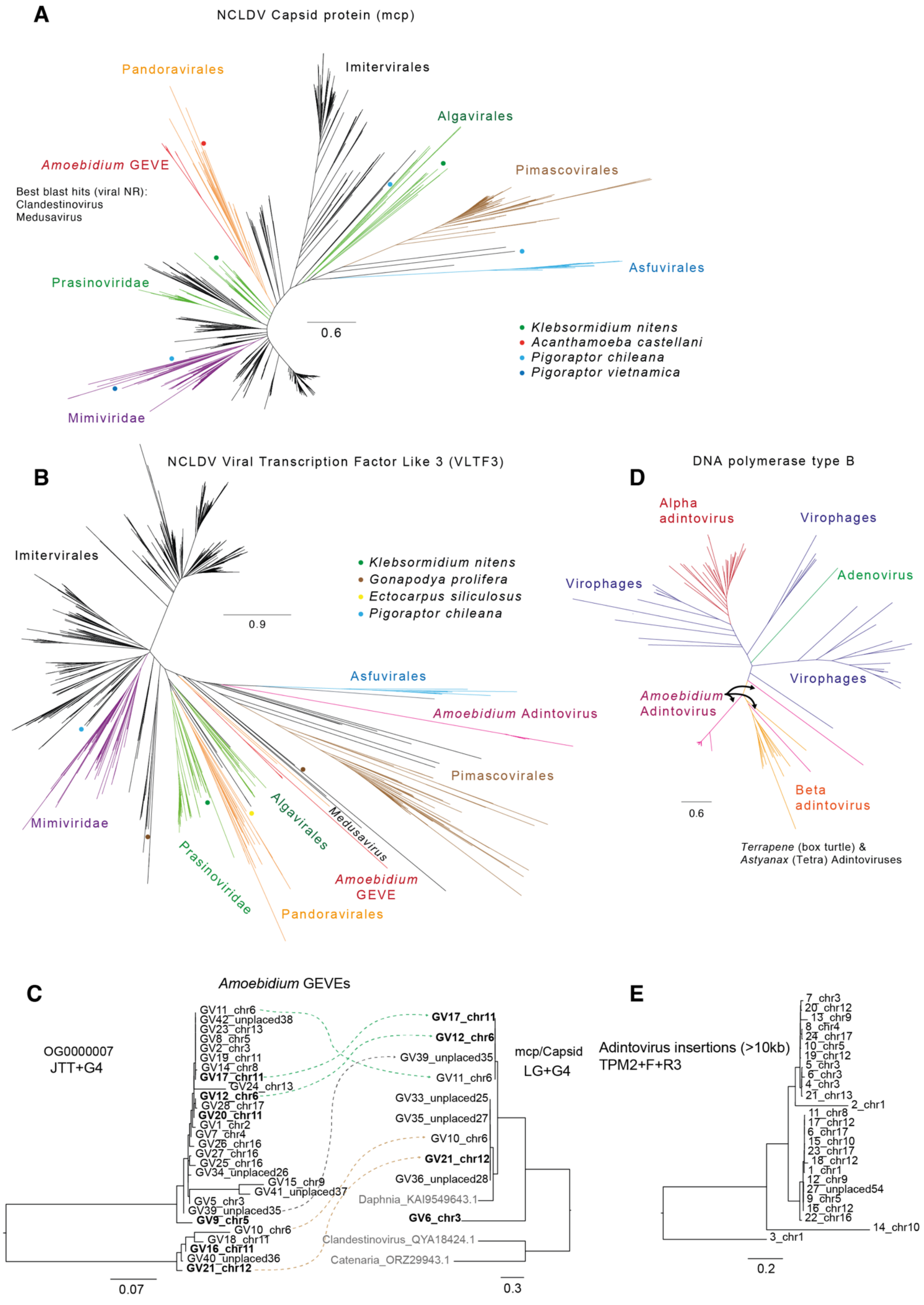

A
NCLDV Capsid protein (mcp)
Pandoravirales
Imitervirales
Algavirales
Pimascovirales
Amoebidium GEVE
Best blast hits (viral NR):
Clandestinovirus
Medusavirus
Prasinoviridae
Asfuvirales
0.6
Klebsormidium nitens
Acanthamoeba castellani
Pigoraptor chileana
Pigoraptor vietnamica
Mimiviridae
B
NCLDV Viral Transcription Factor Like 3 (VLTF3)
Imitervirales
Klebsormidium nitens
Gonapodya prolifera
Ectocarpus siliculosus
Pigoraptor chileana
0.9
Asfuvirales
Amoebidium Adintovirus
Pimascovirales
Mimiviridae
Algavirales
Medusavirus
Prasinoviridae
Amoebidium GEVE
Pandoravirales
D
DNA polymerase type B
Alpha adintovirus
Virophages
Virophages
Adenovirus
Virophages
Amoebidium Adintovirus
Beta adintovirus
0.6
Terrapene (box turtle) &
Astyanax (Tetra) Adintoviruses
C
Amoebidium GEVEs
OG0000007
JTT+G4
mcp/Capsid
LG+G4
GV11_chr6
GV42_unplaced38
GV23_chr13
GV8_chr5
GV2_chr3
GV19_chr11
GV14_chr8
GV17_chr11
GV24_chr13
GV12_chr6
GV28_chr17
GV20_chr11
GV1_chr2
GV7_chr4
GV26_chr16
GV27_chr16
GV25_chr16
GV34_unplaced26
GV15_chr9
GV41_unplaced37
GV5_chr3
GV39_unplaced35
GV9_chr5
GV10_chr6
GV18_chr11
GV16_chr11
GV40_unplaced36
GV21_chr12
0.07
GV17_chr11
GV12_chr6
GV39_unplaced35
GV11_chr6
GV33_unplaced25
GV35_unplaced27
GV10_chr6
GV21_chr12
GV36_unplaced28
Daphnia_KAI9549643.1
GV6_chr3
Clandestinovirus_QYA18424.1
Catenaria_ORZ29943.1
0.3
E
Adintovirus insertions (>10kb)
TPM2+F+R3
7_chr3
20_chr12
13_chr9
8_chr4
24_chr17
10_chr5
19_chr12
5_chr3
6_chr3
4_chr3
21_chr13
2_chr1
11_chr8
17_chr12
6_chr17
15_chr10
23_chr17
18_chr12
1_chr1
12_chr9
27_unplaced54
9_chr5
16_chr12
22_chr16
14_chr10
3_chr1
0.2

Fig. S5.

Phylogenetic alliances of *Amoebidium* endogenized viruses. (**A**) Maximum-likelihood phylogeny of the NCLDV major capsid protein (PF04451) found in the Giant Virus Database, including select GEVEs. *Amoebidium* sequences are found in the red clade embedded in the *Pandoravirales* clade. Potentially endogenized capsid proteins in the filastereans *Pigoraptor chileana/vietnamica*, algae *Klebsormidium nitens* and the amoebozoan *Acanthamoeba castellanni* displayed with coloured dots. (**B**) Maximum-likelihood phylogeny of the VLTF3 (Pox_VLTF3, PF04947) genes found in the Giant Virus database and select eukaryotes. *Amoebidium* GEVEs are shown in a red branch sister group to *Pandoravirales*, including *Medusavirus*. *Amoebidium* adintoviruses are shown in purple, no other polinton-like viruses are shown as these do not normally encode VLTF3, which was probably acquired from a giant virus. (**C**) Amino acid maximum-likelihood phylogeny of a widely distributed Orthogroup (OG000007) across GEVEs, and major capsid proteins with outgroups from panel A. Each GEVE insertion is named as GV[number], and their chromosome of origin. Dotted lines connect insertions that encode both ORFs. (**D**) Maximum-likelihood phylogeny of DNA polymerase type B ORF from adintoviruses, adenoviruses and virophages from (39). (**E**) Nucleotide maximum-likelihood phylogeny of full-length adintovirus insertions. These are labelled with numbers reflecting chromosome of origin. Bars depict the substitutions per site in all phylogenetic trees.

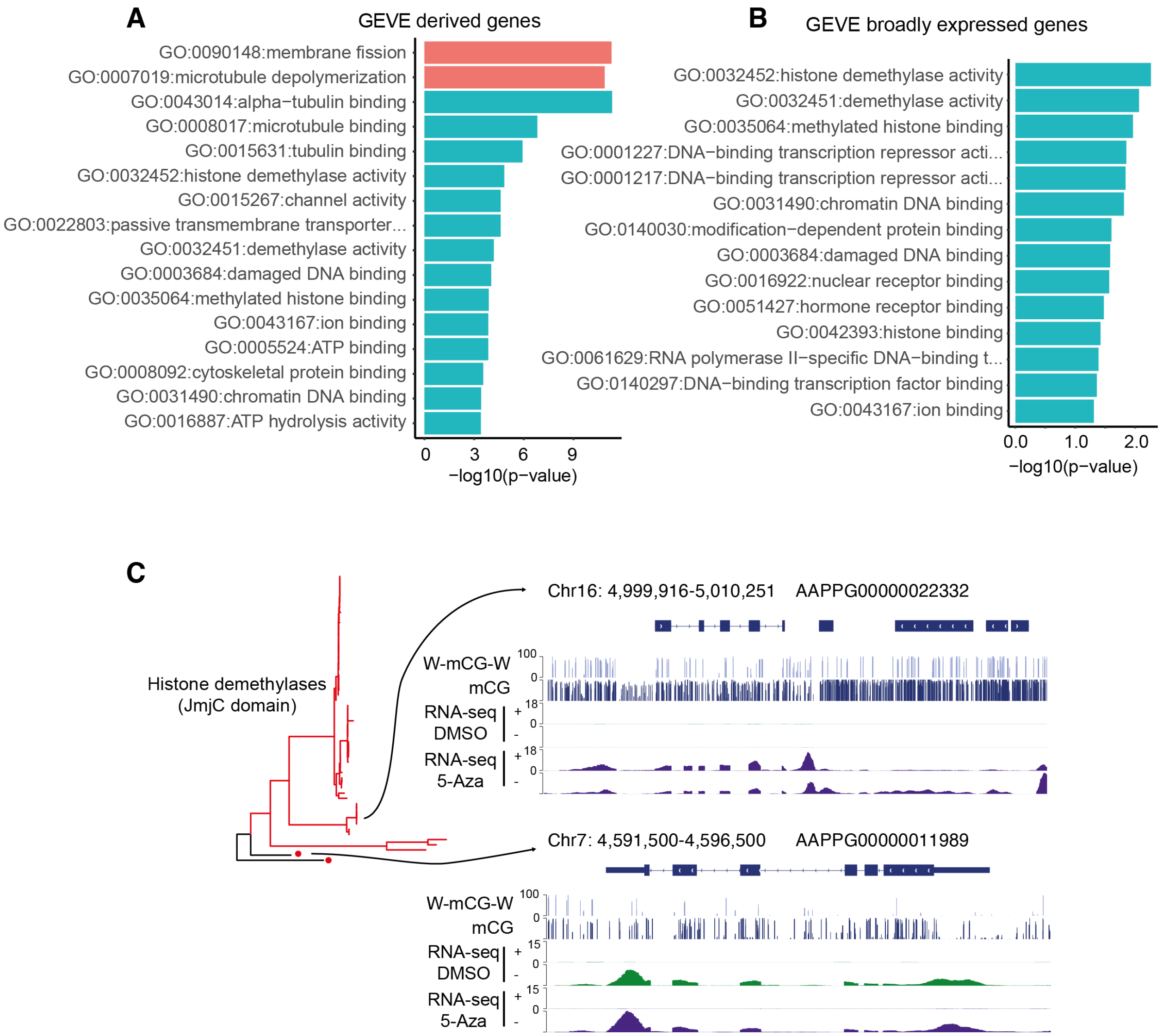

Fig. S6.

Giant virus-derived genes are enriched for histone-demethylase activity. (**A**) Gene Ontology enrichment for all genes encoded in GEVEs with the background of all *Amoebidium* genes. Red bars indicate “Biological Process” GO categories, while turquoise bars indicate “Molecular Function” GOs. (**B**) Gene ontology enrichment of genes encoded in GEVEs that are transcribed in culture conditions (TPM >1). (**C**) Genome browser snapshots of KDM4-like genes found in GEVEs (chr16, high [ATC]mCG[ATG] methylation) or found as host genes (chr 4, lack of [ATC]mCG[ATG] methylation). RNA-seq tracks are divided per strand (positive or negative) and display control and 5-Azacytidine treatment conditions described in **Fig. 3A**.

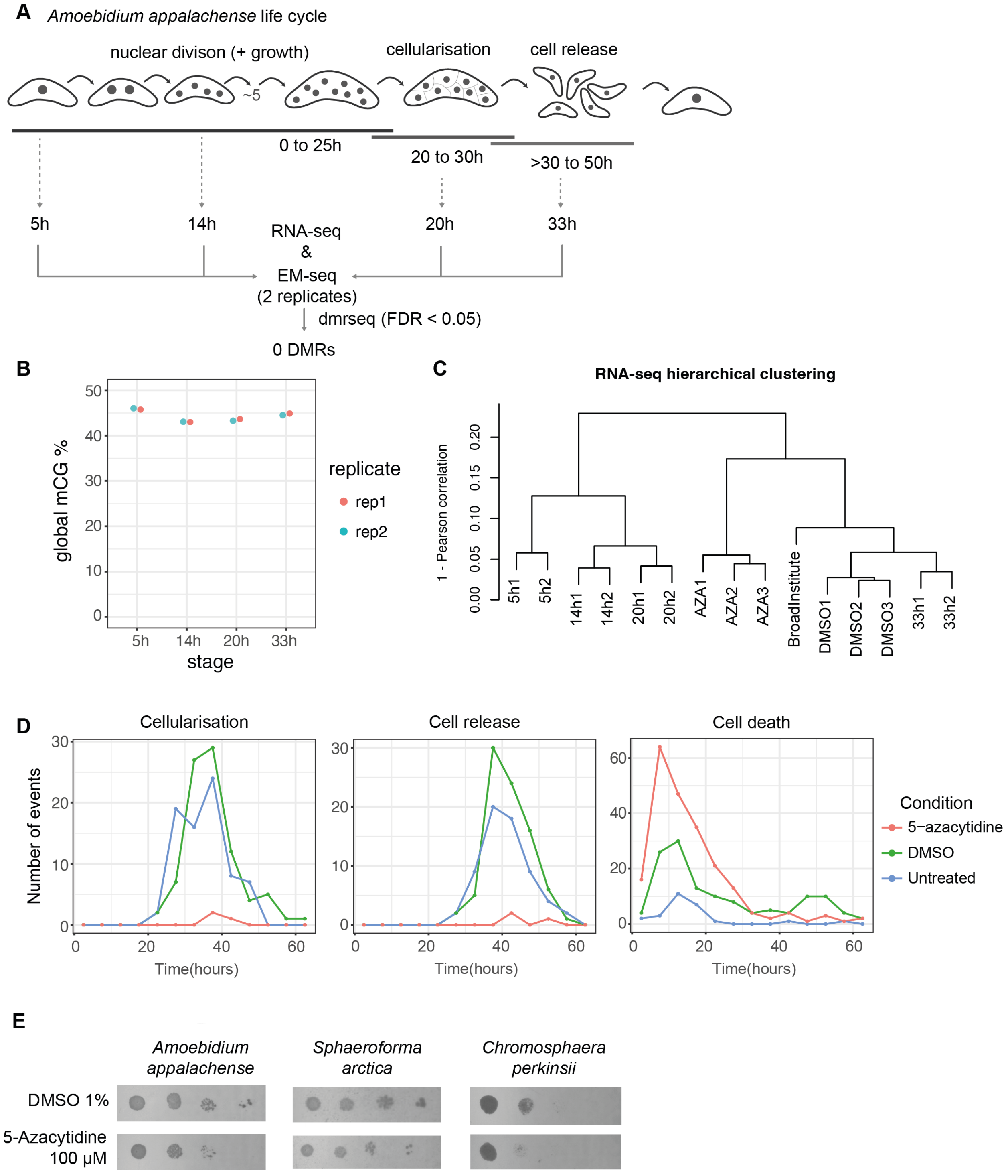

Fig. S7.
***Amoebidium* developmental methylation dynamics and response to 5-Azacytidine treatment**. (**A**) Scheme showing the average timings of *Amoebidium appalachense* coenocytic life cycle. Nuclear divisions within a coenocyte last for about 25 hours, reaching 16 to 32 nuclei.

Cellularization happens around 20h post culture start, and cellular release past 30h. Sampled time points for RNA-seq and Enzymatic Methyl-seq are shown. The R package dmrseq was used to identify differentially methylated regions (DMRs) across developmental stages, finding 0 DMRs passing the non-conservative FDR threshold of 0.05. (**B**) Global CG methylation levels of developmental Enzymatic Methyl-seq replicates. (**C**) Hierarchical clustering of RNA-seq samples of developmental and control (DMSO)/5-Azacitidine (AZA) treatments clustered by Pearson correlation distance calculated in R. (**D**) Distribution of cellularisation, cellular release and spontaneous death events recorded in live microscopy videos of *Amoebidium appalachense* cultures grown in 10% BHI media, 10% BHI with DMSO at 1% and 10% BHI with 5-Azacytidine at 100 uM. (**E**) Agar plates showing *Amoebidium* colony growth in 10% BHI or *Sphaeroforma* and *Chromosphaera* in Marine Broth media upon DMSO and 5-Azacytidine treatments. Left to right: 5µl 1x, 10x, 100x, 1,000x and 10,000x serial dilutions of saturated culture.

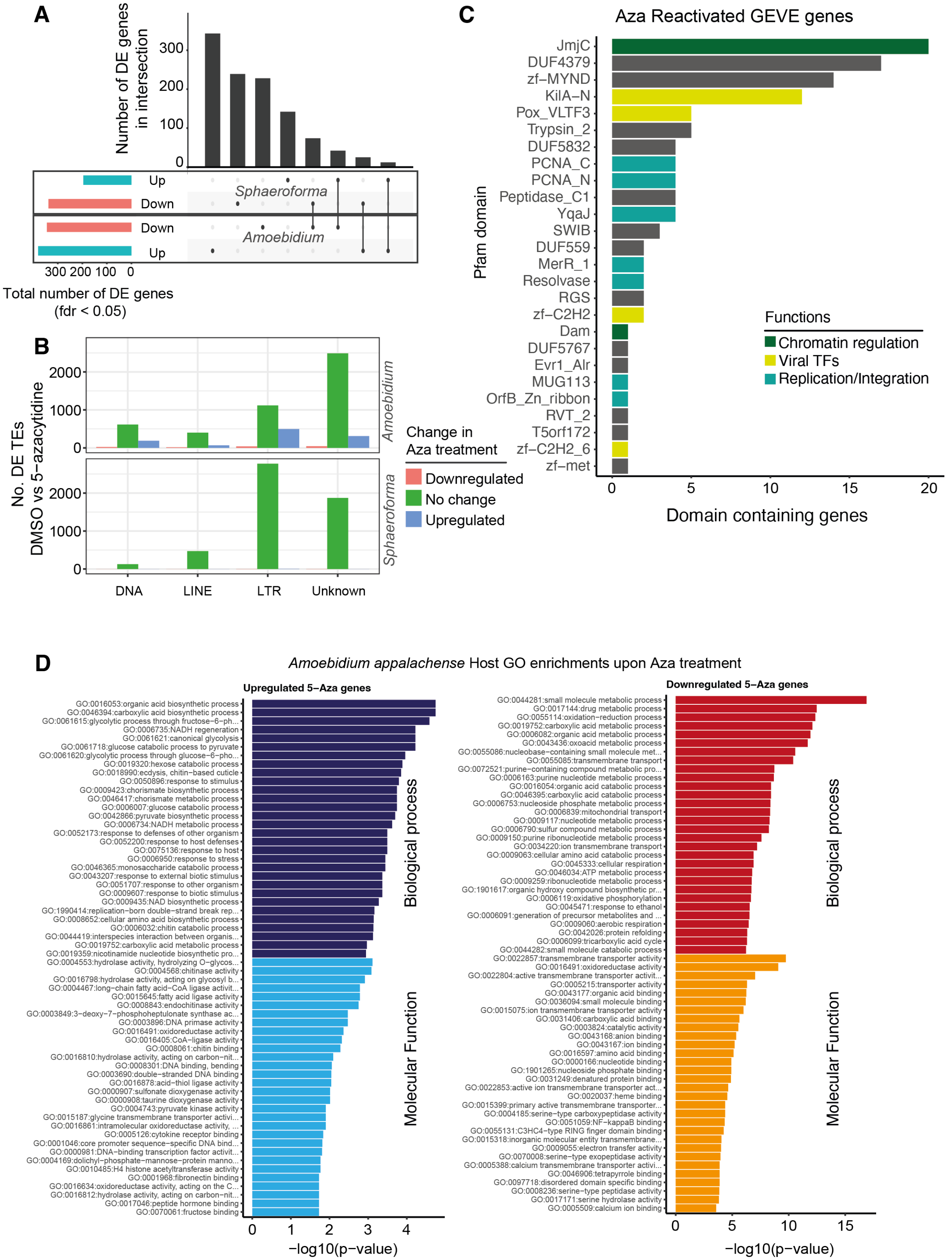
A
Number of DE genes in intersection
300
200
100
0
Up
Down
Sphaeroforma
Down
Amoebidium
Up
300 200 100 0
Total number of DE genes (fdr < 0.05)
B
No. DE TEs DMSO vs 5−azacytidine
2000
1000
0
Amoebidium
Sphaeroforma
DNA
LINE
LTR
Unknown
Change in Aza treatment
Downregulated
No change
Upregulated
C
Aza Reactivated GEVE genes
Pfam domain
JmjC
DUF4379
zf−MYND
KilA−N
Pox_VLTF3
Trypsin_2
DUF5832
PCNA_C
PCNA_N
Peptidase_C1
YqaJ
SWIB
DUF559
MerR_1
Resolvase
RGS
zf−C2H2
Dam
DUF5767
Evr1_Alr
MUG113
OrfB_Zn_ribbon
RVT_2
T5orf172
zf−C2H2_6
zf−met
0
5
10
15
20
Domain containing genes
Functions
Chromatin regulation
Viral TFs
Replication/Integration
D
Amoebidium appalachense Host GO enrichments upon Aza treatment
Upregulated 5−Aza genes
Biological process
Molecular Function
0
1
2
3
4
−log10(p−value)
Downregulated 5−Aza genes
Biological process
Molecular Function
0
5
10
15
−log10(p−value)

Fig S8.

Transcriptional response to 5-Azacytidine in *Amoebidium* and *Sphaeroforma*. (**A**) Upset plot showing the amount of differentially expressed (DE) genes (DEseq2, one to one orthologues) shared across *Amoebidium appalachense* and *Sphaeroforma arctica* comparing DMSO and 5-Azacytidine. (**B**) Transcriptional response of TE individual insertions to 5-Azacytidine treatment in *Amoebidium* and *Sphaeroforma* measured by TElocal. Only TEs larger than 500 bp were included. "No change" indicate TEs which do not change expression level (fdr > 0.05, DEseq2 package) or are silenced throughout. (**C**) Most abundant Pfam domains encoded in GEVE genes reactivated upon 5-Azacytidine treatment. (**D**) Gene ontology enrichments of *Amoebidium* host genes upregulated and downregulated upon 5-Azacytidine treatment (p-value as obtained from TopGO package using default parameters).

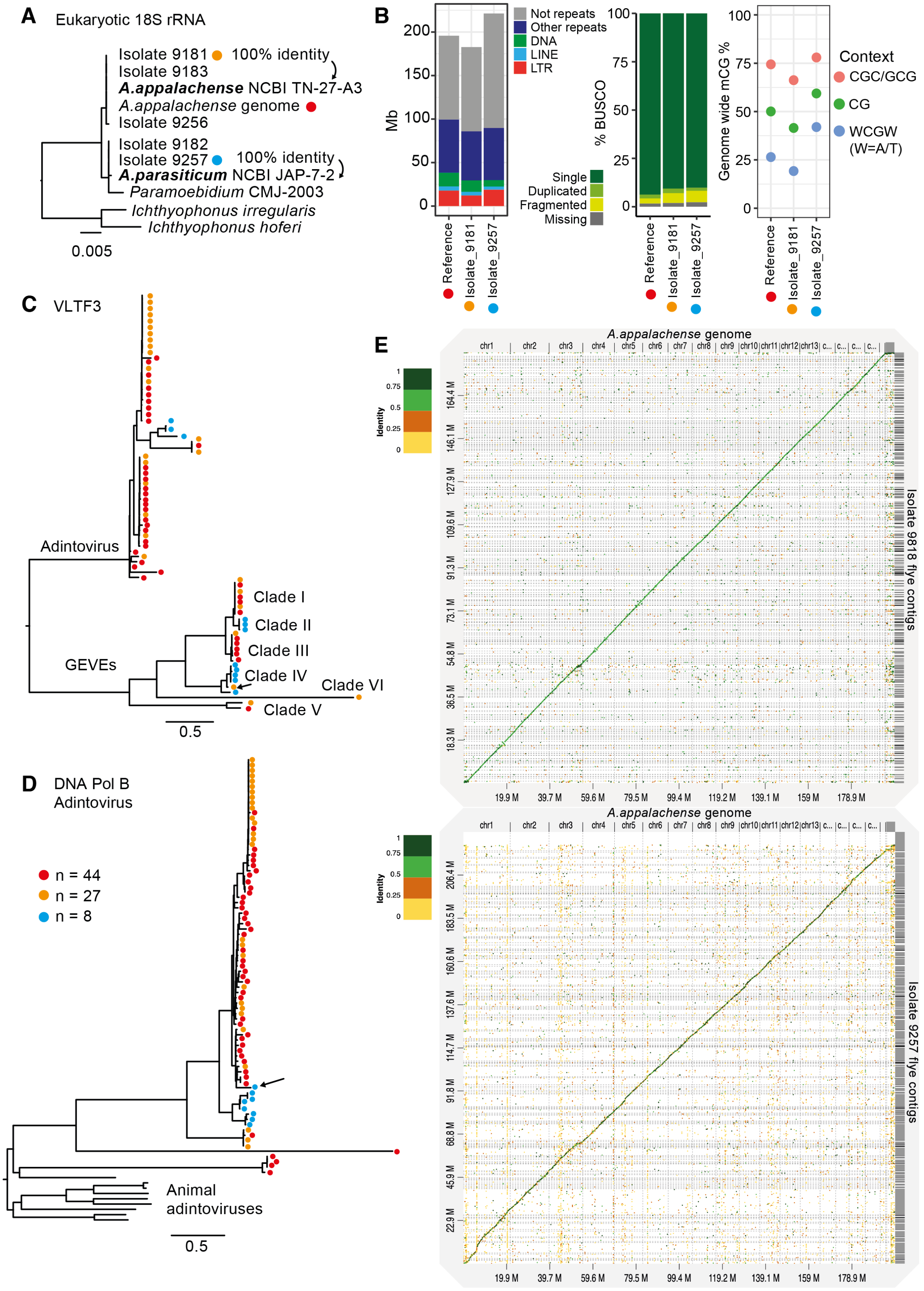
A
Eukaryotic 18S rRNA
Isolate 9181
100% identity
Isolate 9183
A.appalachense NCBI TN-27-A3
A.appalachense genome
Isolate 9256
Isolate 9182
Isolate 9257
100% identity
A.parasiticum NCBI JAP-7-2
Paramoebidium CMJ-2003
Ichthyophonus irregularis
Ichthyophonus hoferi
0.005
B
Mb
Not repeats
Other repeats
DNA
LINE
LTR
% BUSCO
Single
Duplicated
Fragmented
Missing
Genome wide mCG %
Context
CGC/GCG
CG
WCGW
(W=A/T)
Reference
Isolate_9181
Isolate_9257
C
VLTF3
Adintovirus
GEVEs
Clade I
Clade II
Clade III
Clade IV
Clade VI
Clade V
0.5
D
DNA Pol B
Adintovirus
n = 44
n = 27
n = 8
Animal
adintoviruses
0.5
E
A.appalachense genome
Identity
Isolate 9818 flye contigs
A.appalachense genome
Identity
Isolate 9257 flye contigs

Fig. S9.

Genomes and endogenized viral phylogenies of *Amoebidium* isolates. (**A**) Maximum likelihood phylogenetic tree displaying the nuclear ribosomal 18S RNA phylogeny of *Amoebidium* isolates and closely related *Ichthyophonus* and *Paramoebidium* genus. The Isolate 9181 (orange) 18S is 100% identical to *A. appalachense* in NCBI and Isolate 9257 (blue) 18S is 100% identical to *A. parasiticum* in NCBI. (**B**) Genome size, repeat distribution, BUSCO completeness and global CG methylation levels across the 3 *Amoebidium* genomes. (**C**) Maximum likelihood phylogeny of VLTF3 encoded in giant virus and adintovirus endogenization events. Dots represent the genome they come from following panel **Fig. 4A** colour code. (**D**) Maximum likelihood phylogeny of DNA Polymerase family B encoded in adintoviruses. Coloured dots represent genome of origin. (**E**) Dot plot showing the genome alignment of contig level assemblies of Isolate 9181 and 9257 compared to the chromosome scale *A. appalachense* reference genome. Identity of alignment windows is calculated with minimap2 (with the D-GENIES server).

Supplementary Figure Captions

Supplementary Table S1. Number of tracked cells for growth experiments.
Supplementary Table S2. Oxford Nanopore sequencing statistics for all the *Amoebidium* strains
Supplementary Table S3. List of RNA amounts used for mRNA-seq library constructions.
Supplementary Table S4. EM-seq libraries coverage and statistics.
Supplementary Table S5. Database of species screened for DNMTs, GEVEs and giant virus markers.
